# Technology-Enhanced Health Care in Smart Homes: Scoping Review of Sensor Technologies, Clinical Applications, Integration Challenges, and Future Directions

**DOI:** 10.2196/89189

**Published:** 2026-09-30

**Authors:** Yomna El-Saboni, Samira Awwal, Rakesh Mishra, Nityanand Sharma, Anshu Gaur, Leigh Fleming

**Affiliations:** 1 Department of Engineering, School of Computing & Engineering, University of Huddersfield, Queensgate, Huddersfield, England, HD1 3DH, United Kingdom, 44 7575606030

**Keywords:** technology-enhanced health care (TEH), smart homes, Internet of Medical Things (IoMT), remote health monitoring, wearable sensors, adaptable analytics, independent living, interoperability, mobile phone

## Abstract

**Background:**

Smart home technologies integrated with technology-enhanced health care (TEH) systems are transforming residential care by supporting independent living, continuous health monitoring, and remote clinical interventions. The Internet of Medical Things, wearable biosensors, and AI-driven analytics enable proactive health care delivery and personalized interventions, particularly for older adults and individuals with chronic conditions.

**Objective:**

This review synthesizes literature on TEH integration within smart homes, examining global deployment patterns, technological maturity, biomedical sensor integration, machine learning applications, and health outcomes. It also identifies implementation challenges and disparities to improve digital health care strategies.

**Methods:**

A scoping review was conducted across PubMed, Scopus, Web of Science, ScienceDirect, and IEEE Xplore for peer-reviewed studies published between January 2005 and February 2025. Following screening of 6276 records, 169 studies were included, covering experimental, qualitative, and system design methodologies. Data were extracted on geographic deployment, sensor types, TEH architectures, machine learning algorithms, clinical outcomes, and adoption barriers.

**Results:**

TEH adoption is concentrated in Europe, East and Southeast Asia, and higher-income countries, with potential emerging initiatives in West Asia in lower-income regions. Smart home maturity ranges from foundational systems with basic automation to connected ecosystems with centralized Internet of Things coordination, and intelligent systems with data-driven adaptive monitoring. The literature was synthesized across thematic domains, including sensor technologies, smart home infrastructure, predictive analytics, telehealth integration, and ethical and regulatory considerations, and interpreted through a 3-level maturity taxonomy of foundational, connected, and intelligent smart home systems. Integration of biomedical sensors can enable continuous monitoring of cardiovascular, respiratory, neurological, metabolic, and mobility parameters, while machine learning algorithms can support early disease detection, predictive health analytics, activity recognition, and personalized interventions. Evidence from current literature indicates remote monitoring improves early detection of health issues, chronic disease management, medication adherence, and psychological well-being. Several studies reported that remote monitoring systems improved early detection of health deterioration, chronic disease management, medication adherence, and patient well-being. Adoption barriers include interoperability challenges, data privacy, digital literacy gaps, social and economic disparities, and long-term sustainability concerns.

**Conclusions:**

Unlike previous work, this review emphasizes practical adoption barriers, interoperability challenges, and equity considerations alongside sensor performance and system integration. The key significance in this scoping review is how it highlights technological trends and implementation patterns while mapping global deployments of TEH smart homes through encompassing wearable and environmental systems. It demonstrates realistic integration scenarios and how it enhances independent living, preventive care, and personalized health management while reducing hospitalizations and health care costs. It demonstrates how widespread implementation requires standardized evaluation frameworks, robust interoperability, adaptable design, equitable access, and clinically friendly integration. By addressing technical, social, and regulatory challenges, smart home systems can achieve scalable, sustainable, and effective digital health care delivery.

## Introduction

### Rationale

The delivery of health care services has undergone a significant transformation in recent years, driven by the growing demand for personalized, preventive, and accessible care [1]. This ongoing transformation was further accelerated by the COVID-19 pandemic, which heightened public awareness of continuous health monitoring and expanded attention beyond older adult populations to include younger individuals, particularly those exposed to occupational stress and long working hours. In both high-income and resource-limited communities, this shift underscores the urgent need for early-stage disease detection and proactive health management. Early intervention not only improves clinical outcomes but also empowers individuals, including those with disabilities, to manage their health through digital platforms and remote care technologies [2,3].

Technology-enhanced health care (TEH) systems integrated within smart home environments offer a promising solution to the growing demand for personalized and proactive care. Smart homes, equipped with high-speed internet and embedded with sensors, smartphones, wearable devices, and intelligent software, provide a robust infrastructure for continuous health monitoring [4]. These environments can seamlessly incorporate a wide range of biomedical sensors such as heart rate monitors, peripheral capillary oxygen saturation (SpO_₂_) sensors, breath analyzers, skin conductance sensors, glucose monitors, etc, into daily living spaces, enabling real-time data collection and personalized health interventions [5]. TEH systems leverage wearable technologies, mobile apps, and edge computing to facilitate disease prediction, trend analysis, and remote diagnostics, thereby enhancing health care delivery and reducing the burden on clinical professionals [6,7].

Compared to conventional telehealth models and stand-alone digital health interventions such as mHealth (mobile health) applications or remote monitoring devices, TEH integration offers superior data accessibility, contextual awareness, and continuous monitoring. Health data collected within smart homes can trigger physician engagement when early signs of disease progression are detected, while also supporting personalized recommendations for diet, exercise, and lifestyle modifications. Furthermore, the integration of sensor networks and smart devices enables the development of digital twinning (which is a virtual representation of smart homes that simulate residents’ behaviors, health status, and environmental conditions). These digital twins allow for predictive modeling of disease progression, early detection of health risks, and optimization of health care resources, thereby supporting proactive and personalized interventions that enhance patient outcomes and system efficiency [8].

The COVID-19 pandemic revealed critical vulnerabilities in global health care systems, including delays in vaccine distribution and overburdening of medical infrastructure [9]. In such a context, TEH integration within residential environments plays a vital role in minimizing the need for physical clinic visits, facilitating remote patient management, and enabling timely medical intervention. TEH systems can assist public health authorities in predicting disease spread and progression based on demographic, behavioral, and environmental factors, thereby supporting targeted containment and mitigation strategies.

At the core of TEH systems are Internet of Things (IoT)–based health sensors, which can be seamlessly integrated into existing smart home networks. Data collected from these sensors is transmitted to smart devices and health care applications, where edge computing platforms perform preliminary processing before forwarding relevant information to cloud servers and health care providers [10]. The deployment of TEH within smart homes requires a multidisciplinary approach to address challenges related to data acquisition, storage, compression, networking, and analytics. Such technologies allow health practitioners to observe parameters such as irregular activities, sleep patterns, high heart rate, or sudden changes in weight remotely and in real time, which can help detect early stages of a health crisis [11].

Given the potential of TEH-smart home integration to improve health outcomes and promote independent living, it is essential to understand the current landscape of TEH technologies, including sensor systems, wearable devices, and smart home infrastructures, and how they are implemented and evaluated. This review presents a systematic investigation into both the integration of TEH systems with smart homes and the use of sensors and monitoring devices, aiming to identify existing gaps and opportunities for improvement. Of 169 peer-reviewed papers analyzed from multiple databases, this study stands out by highlighting the need for a clear framework for assessment and evaluation of TEH interventions. The findings are intended to inform civil, mechanical, electronic, and computing engineers, biosensor engineers, policymakers, and industry stakeholders in developing strategies for effective TEH integration, ultimately contributing to inclusive, scalable, and clinically robust smart health care environments.

### Objectives

The main objectives of this paper are summarized in the following points: (1) Synthesize current evidence on TEH integration in smart homes, including global deployment patterns, technological maturity, performance of biosensors, and AI-driven health analytics. (2) Identify adoption barriers and evaluate their impact on user acceptance and long-term efficient adoption; which investigates primarily interoperability, usability, and regulatory frameworks. (3) Highlight technological trends, practical implementation scenarios, and implications for future research in medical smart homes and clinically effective smart home health care solutions.

The remainder of this paper is structured as follows: the methodology section details the systematic review approach, including search strategy, selection criteria, and qualitative analysis methods; this is followed by Results, Discussion, and Conclusions sections, which synthesize current evidence, identify research gaps, and provide recommendations for future TEH-smart home integration initiatives.

## Methods

### Study Design

This study was conducted as a scoping review to map, examine, and synthesize the breadth of existing literature on the integration of TEH within smart home environments. A scoping review approach is appropriate for this topic due to the heterogeneity of study designs, technologies, clinical applications, and evaluation methods, as well as the rapidly evolving nature of digital health and smart home research.

This review was conducted in accordance with the methodological framework originally proposed by Arksey and O’Malley [12] and subsequently refined by Levac et al and Peters et al [13,14]. Reporting followed the PRISMA-ScR (Preferred Reporting Items for Systematic Reviews and Meta-Analyses extension for Scoping Reviews) guidelines [15] to ensure transparency, reproducibility, and methodological rigor. A PRISMA-ScR flow diagram was used to document the study identification and selection process. The principles of rapid evidence assessment informed the efficiency of the search process, particularly in managing a broad multidisciplinary evidence base [16]. The current review is explicitly positioned as a PRISMA-ScR–compliant scoping review. The objective was to map evidence, identify key concepts, evaluation approaches, and research gaps, rather than to assess intervention effectiveness or conduct formal risk-of-bias appraisal.

This comprehensive review adopts a structured five-stage approach to ensure transparency, reproducibility, and analytical rigor in exploring TEH integration within smart home environments. The first stage involves research objectives and guiding questions formulation to establish scope and relevance. The second stage involves developing a search strategy, including the use of electronic databases, targeted keywords, and Boolean logic to capture relevant literature. Third, eligibility criteria are defined, specifying inclusion and exclusion parameters based on population, intervention type, setting, publication date, and language to ensure consistency in paper selection. Fourth, the screening and selection process is conducted, applying the eligibility criteria systematically. Finally, a qualitative synthesis was conducted to investigate the selected literature in relation to key aspects, including sensor technologies, interoperability frameworks, system performance, user experience, and ethical considerations. This multidimensional evaluation enabled a detailed study of how TEH systems are designed and implemented in independent residential environments, providing insight into both their technical feasibility and measurable health care impact.

### Research Questions

The primary objective of this scoping review was to map the scope, characteristics, and evaluation approaches of TEH systems integrated within smart home environments, with regard to technical architectures, clinical applications, sociotechnical factors, and reported health outcomes. The following questions guided this review:

1. To what extent, and through which mechanisms, do TEH sensing systems integrated within smart home environments support health care delivery, preventive care, and healthier living conditions?
2. How are data transfer, system interoperability, resource allocation, and operational efficiency addressed within TEH-enabled smart homes to support scalable and sustainable health care solutions?
3. Which assessment tools and evaluation methodologies are used to measure the performance, usability, and clinical impact of TEH integration, and how do sociotechnical factors such as usability, accessibility, digital literacy, and trust influence their adoption?

These questions were intentionally broad, consistent with scoping review guidance, to capture the full range of technologies, study designs, and evaluation approaches present in the literature.

### Search Strategy

A structured electronic literature search was conducted across 6 major academic databases: Web of Science, Scopus, PubMed, IEEE Xplore, ScienceDirect, and Google Scholar. The final search was completed in February 2025. Only peer-reviewed journal papers were considered to ensure academic rigor and methodological transparency.

The search strategy combined fixed keywords using Boolean operators ("AND," "OR") and was designed to capture literature spanning technological, clinical, evaluative, and sociotechnical dimensions of TEH-enabled smart homes. Four thematic clusters were organized for search terms presented in Table 1.

**Table 1.** Thematic classification of keywords used for literature search across smart environments, TEH^a^, evaluation approaches, and sociotechnical adoption factors.

Theme	Keywords
Theme 1: technology and smart environment terms	Smart home, smart house, smart building, automated home, intelligent home, smart technology, connected device, IoT^b^, IoMT^c^, wearable devices, digital health technology, health monitoring devices
Theme 2: health care and TEH terms	Technology-enhanced health care, technology-enabled health care, digital health care, mHealth^d^, telehealth, telemedicine
Theme 3: evaluation and assessment terms	Evaluation framework, assessment tool, clinical evaluation, usability testing, performance metrics, benchmarking, effectiveness, impact assessment
Theme 4: sociotechnical and adoption terms	Usability, accessibility, digital literacy, trust, adoption, sociotechnical, patient outcomes, care pathways

a TEH: technology-enhanced health care.

b IoT: Internet of Things.

c IoMT: Internet of Medical Things.

d mHealth: mobile health.

This strategy ensured comprehensive coverage of the multidisciplinary literature relevant to TEH integration in smart home environments.

### Eligibility Criteria

Eligibility criteria were developed iteratively, consistent with scoping review best practices [14], to balance inclusivity with relevance to this review’s objectives. The final selection of studies was guided by the eligibility criteria summarized in Textbox 1, ensuring alignment with the objectives of this review. Studies were included if they addressed at least one of the following domains:

Regarding core technology, research on smart home technologies, ambient assisted living, IoT, wearable devices, remote patient monitoring, telehealth or telemedicine, mHealth, medical sensor networks, AI, health informatics, or smart environments; these represent the foundational technologies supporting TEH integration in residential settings.

Regarding clinical and disease-specific management, studies focusing on chronic disease management, cardiovascular or respiratory monitoring, diabetes care, dementia support, tuberculosis surveillance, and other home-based clinical applications, these ensure this review captures personalized outcomes and clinical effectiveness.

Regarding smart framework and infrastructure, papers examining integration frameworks, infrastructure design, interconnected systems, smart health care ecosystems, and health data exchange, these criteria reflect structural, interoperability, and system-level considerations critical for effective TEH deployment.

Regarding implementation barriers, studies evaluating technology acceptance, digital literacy, user resistance, privacy and ethical concerns, cost barriers, accessibility, and trust in technology, these criteria ensure that sociotechnical and adoption challenges are systematically captured.

Exclusion criteria removed studies that were irrelevant to smart and remote health monitoring applications, nonpeer-reviewed, or outdated. By explicitly mapping inclusion and exclusion criteria to core technologies, clinical applications, system infrastructure, and implementation challenges, this review maintains methodological rigor, focus, and relevance while following the rapid evidence assessment approach. This eligibility framework justifies the selection of studies by ensuring that only papers contributing meaningful technical, clinical, or sociotechnical insights are included. It also allows this review to systematically identify gaps in evaluation, interoperability, adoption, and scalability, which underpin the strategic recommendations presented later in this paper.

Textbox 1. **Criteria used in the screening process.**

**Inclusion criteria**

- Core technology: smart home technology, ambient assisted living, Internet of Things (IoT), wearable devices, remote patient monitoring, telehealth or telemedicine, mHealth (mobile health), medical sensor networks, AI, health informatics, and smart environments.
- Clinical and disease-specific management: chronic disease management, chronic obstructive pulmonary disease or asthma monitoring, arrhythmia detection, diabetes care, blood pressure monitoring, dementia support systems, tuberculosis surveillance, lung disease diagnostics, and jaundice detection.
- Smart framework and infrastructure: integration framework, infrastructure design, smart health care ecosystem, interconnected systems, and health data exchange.
- Implementation barriers: technology acceptance, digital literacy, user resistance, privacy concerns, cost barriers, demographic challenges, ethical concerns, trust in technology, and accessibility.

**Exclusion criteria**

- Subjective and not peer-reviewed sources: conference abstracts, comments, gray literature, theses, blog posts, editorials, and opinion papers.
- Irrelevant settings: hospital-based care only, intensive care unit (ICU), emergency department interventions, clinical trials without home-based components, gaming technology, and cryptocurrency and blockchain.
- Irrelevant applications: smart homes for energy efficiency only, home automation unrelated to health, and entertainment systems.
- Outdated or nonscalable technologies: obsolete sensor platforms, single-use or nonrechargeable devices, and nondigital interventions.

### Data Charting, Extraction, and Synthesis

Data from the included studies were charted using a structured data extraction framework developed iteratively to ensure consistency and relevance to this review’s objectives. The framework was designed to capture key characteristics across technical, clinical, and sociotechnical dimensions of TEH-enabled smart home systems. Extracted data elements included the following: (1) TEH technology types and sensing modalities; (2) smart home infrastructure components and system integration frameworks; (3) clinical application areas and target populations; (4) evaluation tools, performance metrics, and reported outcome measures; (5) user experience, adoption factors, and sociotechnical considerations; and (6) ethical, privacy, security, and scalability considerations.

This synthesis revealed prominent thematic areas including health care delivery, health monitoring, sensing devices, physical activity, mobility, and medication management. The frequent emphasis on concepts such as monitoring, sensors, connectivity, and intelligent systems indicates that existing TEH-enabled smart home research is largely oriented toward continuous health monitoring, disease detection, and data-driven clinical decision support. These themes informed the organization of results and facilitated the identification of gaps related to evaluation practices, system interoperability, and real-world implementation and adoption.

### Quality Appraisal and Methodological Limitations

In accordance with PRISMA-ScR guidance, this scoping review did not include a formal risk-of-bias or methodological quality appraisal of individual studies [15]. The primary objective of the current review was to map the extent, nature, and characteristics of existing evidence on TEH integration within smart home environments, rather than to evaluate the effectiveness of specific interventions or to compare outcomes across study designs.

There are several methodological limitations. The absence of formal quality assessment means that variation in study rigor, reporting quality, and methodological robustness across included studies was not systematically weighted or quantified. As a result, the synthesis reflects the breadth of available evidence rather than the relative strength of individual findings. The inclusion of diverse study designs limits the ability to draw definitive conclusions regarding clinical effectiveness, comparative performance, or causal relationships between TEH-enabled smart home interventions and health outcomes. The findings should therefore be interpreted as indicative of research trends, dominant approaches, and evidence gaps, rather than as confirmatory evidence of impact. The restriction to English-language, peer-reviewed publications may have resulted in the exclusion of relevant studies published in other languages or reported in gray literature, potentially introducing publication bias. This decision was made to ensure methodological transparency and consistency with this review’s objectives and reporting standards.

Despite these limitations, the scoping review methodology is well-suited to the aims of this study and provides a comprehensive overview of current research on TEH-enabled smart home systems. The findings offer valuable insights into prevailing technologies, evaluation practices, and sociotechnical considerations, while highlighting areas where more rigorous, theory-driven, and outcome-focused research is needed.

## Results

### Selection Process

All retrieved citations were imported into the Rayyan web-based screening platform to support systematic screening and duplicate removal. Duplicate records were identified using Rayyan’s automated functions and manual verification. Title and abstract screening were conducted independently by multiple reviewers to assess relevance to this review’s objectives. Full-text screening was subsequently performed for all potentially eligible studies. Any disagreements regarding study inclusion were resolved through discussion and consensus, consistent with recommended scoping review practices [13].

The screening process resulted in an initial pool of 6276 records, from which 169 peer-reviewed papers met the inclusion criteria and were retained for data charting and synthesis. The complete screening and selection process is illustrated using a PRISMA-ScR flow diagram in Figure 1.

**Figure 1.**
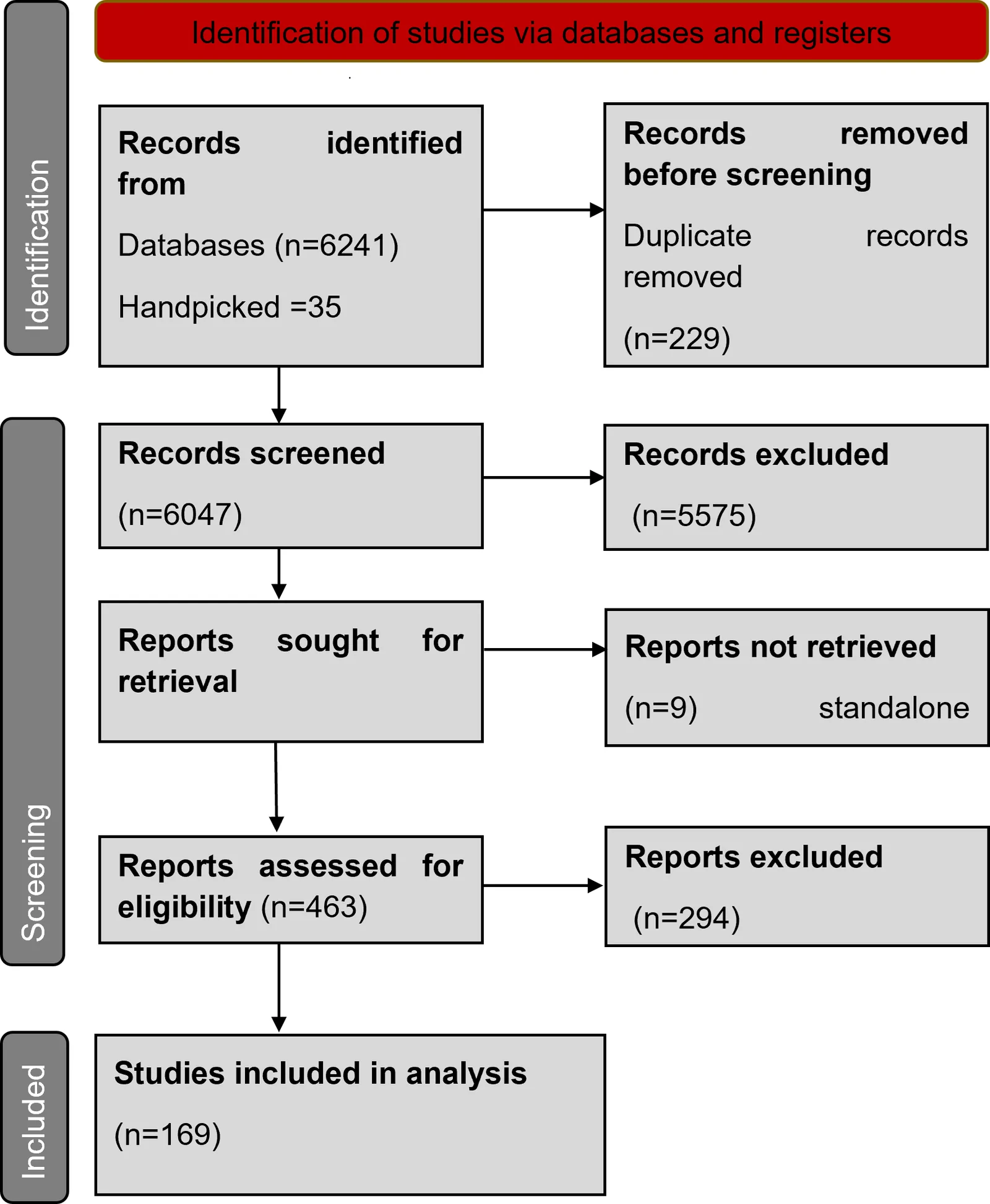
PRISMA-ScR (Preferred Reporting Items for Systematic Reviews and Meta-Analyses extension for Scoping Reviews) flowchart for paper selection following rapid evidence assessment protocol.

### Selection of Sources

The results of this review reflect the literature search identified as examining the integration of telemedicine, environmental monitoring, and smart home technologies for health care applications. Following the screening process, 169 studies met the inclusion criteria and were included in this scoping review. These studies were published between 2005 and 2024, with a noticeable increase in publications after 2010, reflecting the rapid development of IoT architectures, wearable sensors, and AI in health care applications. This literature spans a wide range of interdisciplinary domains, including sensor technologies, environmental monitoring, machine learning (ML)–based health analytics, smart home infrastructures, and telehealth implementation frameworks. While many studies focus on the technological development of sensing and monitoring systems, fewer investigations examine clinical integration, evaluation frameworks, and regulatory considerations, highlighting important gaps in the current literature.

Most papers focused on wearable and ambient sensor technologies, examining health- and environment-affecting factors such as indoor air quality, temperature, and humidity while demonstrating the development of continuous health tracking systems. Another major discipline considered in this review is the infrastructure involved in the design of smart home systems that enables the integration of these sensors. Few studies investigated health prediction analytics and pattern recognition within smart home environments and how it can have an impact on health outcomes. Finally, this review also looks at ethical, regulatory, and usability considerations, interoperability, and evaluation frameworks essential for the effective and responsible deployment of smart home health care technologies. There is a clear gap in regulatory frameworks in the aspect of TEH integration, as shown in Table 2, which requires further development.

**Table 2.** Distribution of the 169 included studies across primary research themes.

Primary research theme	Studies, n (%)
Sensor technologies	68 (40.2)
IoT^a^ infrastructure	34 (20.1)
Machine learning	27 (16.0)
Telehealth	24 (14.2)
Ethics and usability	16 (9.5)
Total	169 (100.0)

a IoT: Internet of Things.

### Characteristics of Source Evidence

#### Demographic Analysis

Smart home technologies have been widely developed and deployed across various European countries, including Germany, Finland, France, Spain, the Netherlands, Sweden, Italy, Ireland, Denmark, Belgium, Greece, Austria, etc. These initiatives predominantly focus on supporting older adult individuals using embedded technologies designed to enhance independent living, safety, and health monitoring [5]. Although the concept of the smart home originated in 1975, the considerable worthwhile transformation in integrated technologies only started within the past 2 decades, with major progress emerging around 2010 in both industry and academic research [17]. Many European nations have since prioritized age-in-place strategies, leveraging TEH systems within residential settings to promote autonomy and reduce dependence on institutionalized care. European institutions have focused on experimental frameworks and pilot studies exploring novel sensing modalities, interoperability standards, and AI-driven data analytics. Examples include research programs funded under the European Union’s Horizon 2020 and Horizon Europe initiatives, which investigate smart environments for older adult care, telemedicine, and cognitive monitoring [18,19]. These projects typically assess feasibility, user experience, and system interoperability on a small to medium scale. In contrast, commercial development has concentrated on impact and scalable implementation. Companies such as Siemens, Bosch, and Philips have integrated TEH features into smart home ecosystems through intelligent lighting, fall detection, and remote health management platforms [20]. This integration required emphasis on new aspects such as data reliability, data privacy compliance, and consumer usability over experimental innovation. They invest in better interoperability with IoT home devices, long-term reliability, and secure integration with health care providers.

The quality and maturity of TEH adoption, however, vary significantly across regions. In the United Kingdom, for instance, notable efforts such as Gloucester’s Smart House and the Cabernets project demonstrated targeted and personalized innovations in dementia-friendly living environments [21,22]. These projects integrate behavioral analytics, motion sensing, and caregiver alert systems to support autonomy and safety. Yet, nationwide scalability remains hindered by interoperability challenges between smart home platforms and health care networks. In East and Southeast Asia, countries such as Japan, South Korea, and Singapore exhibit some of the most mature TEH infrastructures, supported by government-driven aging policies and integration of IoT-based monitoring within residential architecture [23]. These regions have adopted various forms of ambient assisted living and intelligent home automation to elevate the quality of life for aging and vulnerable populations. Similarly, countries in Western Asia, such as Türkiye, Israel, and Saudi Arabia, are in the early to intermediate stages of TEH smart residential adoption that focuses on the development of digital health innovation zones [24]. These initiatives show strong potential for accelerated growth as regulatory frameworks and interoperability standards mature.

The diversity observed in these global patterns indicates that TEH adoption is expanding and reflects a shared commitment to addressing demographic challenges and increasing burden of chronic disease management through innovative smart health care solutions. However, the quality of smart home integration remains uneven. Regions with established regulatory infrastructure, health care digitization, and government support demonstrate higher maturity levels, while developing economies continue to explore foundational frameworks for scalability and affordability. The convergence of scalable research innovation and industry implementation is therefore crucial to achieving sustainable, equitable, and clinically validated TEH smart home ecosystems.

#### Maturity Levels in Smart Home Health Care Ecosystems

Understanding the global variability in TEH adoption provides a contextual foundation for examining how smart home systems evolve in design sophistication. The maturity of health care integration is closely linked to the architectural complexity of smart adaptive ecosystems capable of partial autonomous decision-making. The following section outlines the levels of smart home complexity, illustrating how increasing technological integration enables progressively advanced and personalized health care delivery within residential settings. Smart home environments for health care integration can be conceptualized across 3 progressive levels of complexity: foundational, connected, and intelligent systems [25]. Each level reflects the degree of automation, interoperability, and adaptability embedded within the home infrastructure, which later impacts the scope and sophistication of health care applications that can be supported. Understanding these tiers helps guide the design and deployment of smart home infrastructures aligned with clinical needs, technological capacity, and user readiness.

At the foundational level, smart homes feature basic automation technologies such as smart lighting, thermostats, and motion detectors operating as independent systems. These provide limited yet functional support for simple TEH interventions, including medication reminders, fall prevention lighting, or basic environmental adjustments [26]. Although relatively low cost and easy to implement, foundational systems rely on user activation rather than autonomous health monitoring and thus serve primarily as assistive rather than diagnostic tools. It emphasizes usability and accessibility.

The connected level introduces a wider integrated system through centralized hubs or IoT networks that allow multiple devices to communicate and coordinate actions. This level enables conditional automation and basic data exchange, supporting health workflows such as adjusting air filtration when particulate levels rise, synchronizing temperature controls for patients with chronic respiratory issues, or enabling caregivers to remotely monitor vital signs [27]. Design considerations at this stage emphasize interoperability, reliable data transfer, and secure communication protocols.

Lastly, at the intelligent level, smart homes become adaptive environments that rely heavily on AI, ML, and environmental sensing to autonomously respond to subject needs and physiological cues [28]. These systems interpret biosensor data in real time to optimize environmental conditions, such as adjusting lighting and modifying temperature based on metabolic readings [29]. It can deliver predictive alerts for fall risk, cardiac irregularities, or cognitive decline [30]. Intelligent homes represent the highest maturity in TEH integration, combining predictive analytics, voice and gesture recognition, and automated clinical communication.

#### Integration of Biomedical Sensors With Smart Home Environments

Biomedical sensors represent a core component of smart home systems, enabling continuous monitoring of individual health status and supporting activity recognition in ambient-assisted living contexts [31]. Within these environments, digital sensors facilitate both personalized health tracking, bridging episodic events and chronic condition management. A relevant model outlining the integration of TEH sensors in smart homes for people with mental health problems categorizes the process into four key steps: (1) establishing a digital platform with a selection of biosensors and personalized responsive screen devices, (2) setting up communication links between health care providers and patients, (3) ensuring that end-user requirements are adequately addressed, and (4) engaging stakeholders and collaborators in the development process [30].

Several studies reported computational capacity challenges faced with TEH implementation within residential ecosystems that presented an association with reduced performance in activity recognition and health monitoring systems. For instance, in most health care applications, as shown in the study by Tang et al [32], single-parameter sensors are insufficient to provide a holistic picture of complex patterns, and environmental uncertainty or its uniformity in data collection affects the credibility of predictive models that depend on singular localized feature sets [33]. Therefore, it is important to integrate both ambient and biomedical sensors together to assist in health monitoring decision-making.

A schematic visualization of this concept is presented in Figure 2, illustrating the TEH architecture integrated within a smart home setting. The main objective of integrating TEH architecture in smart homes is that it improves the quality, efficiency, and accessibility of medical services. At its core, the model shown in the figure aims to connect biomedical sensors, home IoT devices, and data processing platforms to form an intelligent, responsive health care network. For example, wearable biosensors such as ECG (electrocardiography) patches and SpO_₂_ monitors continuously capture physiological data, while environmental sensors record temperature, humidity, and air quality. These inputs are then transmitted via connected gateways or Wi-Fi routers to edge or cloud computing platforms. The data is then processed in real time using ML algorithms. Initially, the system provides feedback through user interfaces such as smartphone dashboards, smart mirrors, or voice assistants. Next, it will start alerting users or caregivers to abnormal health trends. In practical implementations, such systems have been used to detect early signs of respiratory distress in patients with chronic obstructive pulmonary disease, identify irregular heart rhythms for arrhythmia prevention, and monitor activity patterns to predict fall risk among older adults. This architecture represents an interconnected ecosystem that links continuous sensing, intelligent analytics, and clinical communication channels to support proactive, adaptive health care within residential settings.

**Figure 2.**
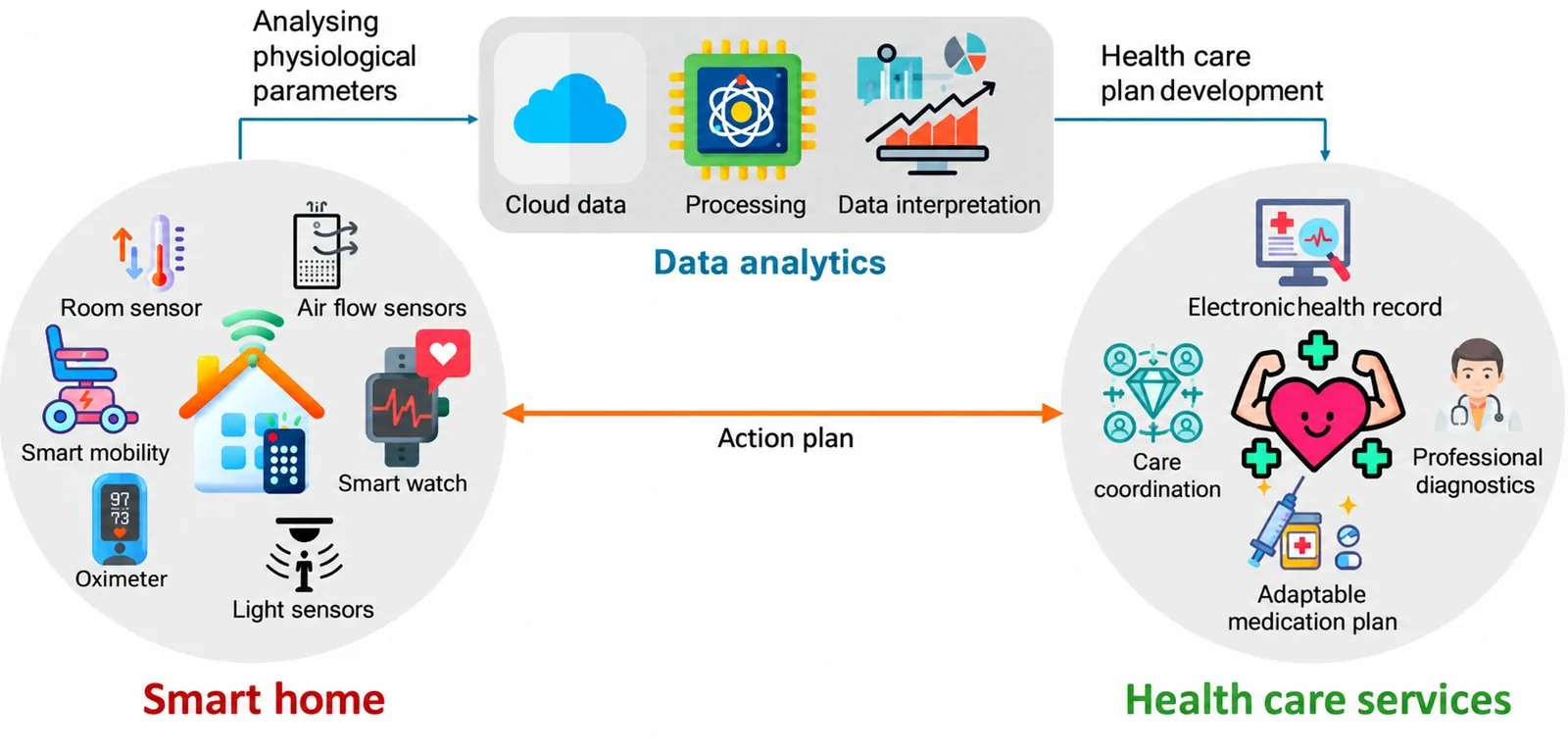
The architecture of a technology-enhanced health care ecosystem illustrates the interaction between biomedical sensors, ambient devices, and technology-enhanced health care processing platforms.

Smart home applications have used sensors to monitor physical movement, glucose levels in patients with diabetes, and patterns of electronic device usage. However, a significant challenge lies in effectively leveraging biomedical sensor data for reliable disease detection and health risk prediction [31,34]. These sensors often generate high-resolution data in large volumes, transmitting information to cloud servers via home networks. This necessitates advanced computational techniques for real-time data processing and analysis. To address these challenges, ML algorithms that involve both supervised and unsupervised learning have been used to interpret sensor data, predict disease onset, and assess disease progression. Effective health prediction models rely heavily on high-quality, longitudinal datasets for training and validation. The availability and diversity of these training datasets directly impact the accuracy and reliability of algorithmic predictions.

Integrating biomedical sensors with ML tools in smart home environments has demonstrated potential to reduce health care costs and alleviate pressure on clinical services by enabling remote diagnostics and early interventions. Therefore, the strategic incorporation of TEH within smart housing infrastructure remains vital for enhancing health outcomes, ensuring proactive care delivery, and supporting independent living for vulnerable populations. The biosensors examined in this review are broadly categorized into 5 distinct groups, each aligned with a specific physiological domain. Examples of each category are summarized in Textbox 1. It demonstrates the broad functional spectrum of biosensors applied within smart home ecosystems. The reviewed devices encompass respiratory, cardiovascular, neurological, biochemical, and movement monitoring applications, using diverse sensing modalities such as radar, optical, biochemical, and mechanical transduction. Integration formats range from wearable patches and textile-integrated sensors to ambient sensors that enable holistic personalized health monitoring. They facilitate continuous, noninvasive data acquisition, supporting early disease detection, specific condition management, and timely adaptive interventions.

The identified literature was organized into thematic domains reflecting the technological and clinical dimensions of TEH-smart home integration, including environmental monitoring, physiological sensing, predictive analytics, health care system interoperability, and regulatory considerations.

#### Wearable Medical Sensors

Wearable biosensors play a critical role in real-time monitoring of physiological parameters within smart home environments. Commonly measured vital signs include heart rate, pulse rate, blood pressure, and blood oxygen saturation (SpO_₂_), which are summarized below in Table 3.

**Table 3.** Examples of biosensor applications commonly used in smart homes.

Application and sensor type	Sensing parameters	Clinical applications
Respiratory monitoring		
Respiration rate monitor [35,36]	Breathing rhythm, inhalation cycles	Asthma, COPD^a^, sleep disorders
RF^b^ sensor [37]	Chest and breathing movement	Lung pulmonary diseases
SpO₂^c^ sensor [38]	Oxygen content in blood	Pulmonary function, COVID-19, hypoxia
Cardiovascular monitoring		
Wearable ECG^d^ sensor [39]	Heart rate, electrical signals	Arrhythmia and stroke detection
Blood pressure monitor [40]	Systolic and diastolic pressure	Hypertension, heart disease
Passive radar system [41]	Chest movement, heart-activity proxy	Nocturnal cardiac monitoring
Mental and neurological monitoring		
EEG^e^ sensor [42]	Brain activity	Seizure detection
Multiarm bandit system [43]	Sleep patterns	Schizophrenia, insomnia
Electronic pill container [44]	Medication, behavioral patterns	Bipolar disorder, cognitive decline
Biochemical and metabolic sensing		
Skin sensor [45]	Glucose concentration	Diabetes management
Sweat sensor [46,47]	Glucose, sleep markers	Diabetes, hydration, sleep
Color, odor, pH sensor [48]	Urinary profile	UTI^f^ detection, hydration analysis
Gas sensor [49]	Air composition (CO^g^, VOCs^h^)	Air quality monitor
Movement and fall detection		
Floor mat [50]	Motion, pressure changes	Activity and mobility tracking for dementia
Watches [51]	Movement, smoke	Fall detection alerts
Electronic textile [52]	Gait, movement, caloric activity	Obesity and daily monitoring
Camera [53,54]	Visual motion capture	Neurodegenerative tracking
Passive radar system [37]	Movement, respiratory proxy signals	Real-time fall and activity detection
Wearable accelerometer [55,56]	Posture	Fall risk, balance monitoring
Smart RFID^i^ sensor [57,58]	Obstacle detection, positional tracking	Fall prevention, assisted navigation

a COPD: chronic obstructive pulmonary disease.

b RF: radio frequency.

c SpO_₂_: peripheral capillary oxygen saturation.

d ECG: electrocardiography.

e EEG: electroencephalography.

f UTI: urinary tract infection.

g CO: carbon monoxide.

h VOC: volatile organic compound.

i RFID: radio-frequency identification.

The sensors presented in Table 3 are particularly significant for a patient’s lifestyle in living with chronic conditions such as heart failure or kidney disease, within the context of smart home environments [59-62]. Other technologies, such as artificial skin implantation, embed biosensors in the body and monitor health indicators under both static and dynamic conditions present substantial impacts on patient independent living [16-18]. Additionally, many wearable platforms are designed to be integrated into clothing, accessories, or adhesive patches, offering unobtrusive monitoring throughout daily life [63]. This has led to advancements in flexible wound monitoring biosensors embedded in wound dressings that facilitate remote automated assessments and treatment adjustments [63]. Another highly effective TEH smart home solution is the development of thin paper sensing strips that are designed to be paired with smartphone interfaces to provide highly accurate and accessible glucose level monitoring [64]. Furthermore, the table illustrates a diverse class of wearable health devices including but not limited to smartwatches, fitness trackers, flexible patches, and voice-activated systems that can acquire physiological data. These data streams can be analyzed using ML techniques to support early disease detection, predictive health monitoring, and personalized care [65]. Many of the monitoring devices mentioned in Table 3 rely on mobile apps to transmit health data from wearable sensors to remote servers, supporting disease management in conditions [66] to enhance self-care outcomes and a more responsive and patient-centric health care model [67]. This resolved the challenge of sending data signals to a local substation. That is because in complex sensor networks, gateway interfaces serve as bridges between sensor nodes and access points, streamlining service delivery and regulating data flow within smart home systems [68]. This integration requires seamless coordination between biomedical informatics and health IT to ensure reliable data acquisition, processing, and communication. A brief illustration of how biomedical data is captured by wearable devices and analyzed is demonstrated in Table 4, which lists the different smartphone software applications used for sensing and communicating different forms of health data.

**Table 4.** Functional classification of TEH^a^ software application in smart home environment.

Category and application	Platform supported	Original health/disease indicator
Voice assistants and smart-home control [69-71]		
Amazon Echo	Bluetooth audio	Diabetes
Google Home	Android (Google LLC)	Glucose levels
OpenHAB	iOS (Apple Inc), Android	Safety for disabled users
Gerikits	iOS, Android	Senior people illness
Health-data platforms [72-76]		
Apple Healthkit	iOS	Diabetes
Samsung Health	Android	Sleep patterns
Fitbit	iOS, Android	Health metrics
Freestyle Libre	iOS	Diabetes
MyFootcare	Android	Foot ulcer
Mental and cognitive health tools [77-81]		
MONARCA	Web-based	Bipolar disorder
FOCUS	iOS, Android	Schizophrenia
CLIN Touch	Mobile	Schizophrenia
Smart FABER	Android	Cognitive impairments
TEH ecosystem [82-84]		
Smart e-Health Gateway	Android	Health monitoring
Care4U	Linux	Health data security
Caregiver App	iOS, Android	Daily activity tracking
Medication management [85]		
iMedBox	Android	Medication management

a TEH: technology-enhanced health care.

The table outlines few medical applications and platforms that underpin TEH integration in smart home environments. The reviewed systems encompass a wide spectrum of digital health tools, including voice-activated assistants, mHealth data platforms, cognitive and mental health monitoring applications, and medication management systems. These technologies operate across major operating platforms such as Android, iOS, and Linux, which demonstrates the need for platform compatibility and ecosystem connectivity. The examples mentioned in the table highlight the transition toward interoperable, adaptable, and case-specific health care solutions, where multimodal data from voice interaction, sensor response, and clinical input converge to enable real-time monitoring, disease management, and personalized health support within domestic settings.

The placement and alignment of sensors on the body are prime factors for achieving optimal system performance. It has been found in studies that increased packet losses have occurred because of network difficulties or misalignment of skin-contact sensors. In addition, some of the wearable devices may be forgotten, removed, or disliked by older adult users, reducing their effectiveness and long-term adoption [42]. The challenge in this type of wearable device is the quality of measurement. Additionally, the data acquired must be verified with conventional sensors to ensure accuracy. These devices are often limited to monitoring only a limited set of physiological parameters, and their constrained computing capability limits the ability to use ML algorithms locally. Furthermore, issues of data latency arise when these devices are used beyond the reliable reach of smart home networks, further complicating real-time health monitoring and timely interventions.

### Biosensor Wearability and Mechanical Flexibility

The convergence of flexible electronics and textile engineering has enabled the development of biosensors that are not only functional but also comfortable, discreet, and adaptable to daily life [86]. Wearable technologies and woven smart textiles can be developed to blend unobtrusively into everyday life, providing continuous monitoring while enhancing comfort and adherence [87,88]. These devices are increasingly designed to conform to the human body’s contours, leveraging materials such as elastomers, conductive inks, and nanofibers to ensure breathability, biocompatibility, and long-term usability.

A compelling example of this innovation is textile-integrated biosensors, as they offer transformative potential for independent living [89]. By embedding sensors into garments or dressings, patients can continuously monitor vital signs, biochemical markers, and environmental conditions without the need for bulky equipment or frequent clinical visits. These systems enable remote health supervision, reduce the burden on caregivers, and empower individuals to manage their health autonomously. The seamless integration of biosensors into textiles achieved through techniques such as screen printing, knitting, and lamination creates what is known as a “second skin” effect, allowing for unobtrusive and personalized health care.

Moreover, the mechanical strain testing of recently developed textile sensors revealed their ability to bend around diameters as small as 1 mm without compromising conductivity, underscoring their suitability for dynamic and health care applications [90]. This level of flexibility not only enhances comfort but also supports continuous data acquisition in real-world environments, a key requirement for smart home integration and patient-centered care.

### Synthesis of Ambient Integrated Sensors

#### Environmental Sensors

Environmental sensors in smart homes are designed to continuously monitor key indoor parameters such as temperature, humidity, and air quality, all of which have a direct impact on human health [91]. These factors are particularly critical for individuals with respiratory conditions such as asthma, chronic obstructive pulmonary disease, and other pulmonary ailments. Variations in these environmental metrics can exacerbate symptoms or trigger acute episodes, making real-time monitoring essential for preventive care and early intervention.

By integrating environmental sensors into smart home ecosystems, it becomes possible to maintain a health-optimized living environment. These sensors detect fluctuations that may compromise air quality, such as increased particulate matter, volatile organic compounds, or elevated humidity, and can trigger automated responses or alerts to mitigate risk. Furthermore, continuous monitoring allows for long-term analysis of environmental factors and their association with health trends, providing insights that can inform early intervention and individualized care. Recent studies have demonstrated the value of such systems in unobtrusive health monitoring and emphasize the role of ambient sensing in private spaces, noting that smart homes equipped with environmental sensors can significantly enhance the management of chronic respiratory diseases by enabling early detection of symptom-related triggers and supporting adaptive interventions [92].

#### Motion and Activity Sensors

Motion and activity sensors play a pivotal role in smart home biosensing systems, particularly for monitoring mobility, detecting falls, and assessing behavioral patterns. Technologies such as smart floor sensors, infrared motion detectors, and accelerometers—often integrated with mobile apps—enable continuous tracking of residents’ movement and activity levels, which is essential for fall detection, gait analysis, and mobility assessment in older adult care settings [93,94]. These sensors can identify deviations in walking patterns or sudden inactivity, which may signal cognitive decline or acute health events [95,96]. The data collected can be analyzed using ML algorithms to detect anomalies, predict falls, and understand individual habits over time, thereby supporting personalized interventions and early diagnosis [55].

Speech-based IoT interfaces further enhance accessibility for individuals with speech and motor disabilities, enabling voice-activated control of smart devices and health monitoring systems. It was previously demonstrated how adaptive speech recognition systems integrated with IoT platforms can empower users with motor impairments to interact with their environment autonomously [97]. Additionally, sensor edge networks can be deployed to localize movement within the home, reducing latency and improving responsiveness. These networks can be paired with anticollision systems that assist individuals with visual impairment by detecting obstacles and providing real-time feedback through auditory or tactile cues.

A sensor edge network could be deployed to track the movement of residents in a smart home environment, and an anticollision system could be paired that would prevent the accidents and provide information of outdoor environment to individuals with visual impairment [98,99].

#### Radar Sensors

Radar sensors offer a compelling solution for contactless health monitoring in smart home environments, particularly when integrated with smart speakers or graphical user interface–based health gadgets. These sensors can detect when a person enters a room or approaches a device, automatically waking it from sleep mode and initiating preprocessing for vital sign measurements such as respiration rate and heart rate. This functionality enhances energy efficiency, supports ambient intelligence, and ensures a seamless user experience.

Recent advancements in radar sensing applications have evolved to include a wide spectrum of health care innovations such as human activity recognition, gait analysis, and vital signs monitoring [100]. They emphasize radar’s ability to detect respiration and heartbeat unobtrusively, making it ideal for smart home integration where passive interaction is key to user comfort and system responsiveness.

Previously, interest has been directed toward validating radar’s capability to monitor multiple individuals’ vital signs in real-world environments [101]. In theory, this system can track movement direction, location, and physiological signals without requiring wearables, reinforcing radar’s role in ambient and predictive health care.

Moreover, innovative approaches have emerged, such as microwave radar embedded in smart furniture, which has demonstrated high precision in monitoring cardiac waveforms and heart rate variability [41]. These systems activate based on user presence and movement, aligning with the concept of radar-triggered device wake-up and automated health data acquisition.

#### Smart Ring Sensing Devices

Smart rings represent a compact and increasingly prominent class of wearable health monitoring devices, designed to track physiological and behavioral parameters comfortably. These rings are equipped with embedded sensors capable of measuring heart rate, heart rate variability, blood oxygen saturation, body temperature, sleep activity and quality, and physical activity levels [102]. By continuously monitoring these biometric indicators, smart rings can support early detection of anomalies, enhance personalized health management, and contribute to improved health outcomes [103].

The device interfaces with a smartphone app that aggregates and analyzes health metrics, enabling users and clinicians to interpret trends in sleep behavior and overall wellness. Advanced sensing techniques within the ring support predictive analytics, offering insights into potential illness onset based on deviations in sleep or activity patterns.

Technically, the smart ring integrates a lithium-ion battery and features enhanced processing capabilities via 6 embedded microcontroller units. These microcontroller units coordinate with multimodal sensors such as accelerometers, PPG (photoplethysmography), and temperature sensors to enable sensor fusion, thereby improving the accuracy and reliability of health data acquisition. This fusion of hardware and software allows the smart ring to function as a powerful tool for continuous, passive health surveillance within smart home ecosystems.

#### Pressure and Vibration Sensors

When embedded in smart flooring systems or furniture, pressure sensors play a critical role in monitoring gait dynamics, postural transitions, and fall events, which are key indicators of mobility and safety in smart home health care environments. These sensors enable spatially resolved pressure mapping, which supports real-time visualization of movement patterns and biomechanical stress [104]. A recent study demonstrated the development of a self-powered smart insole incorporating 22 pressure sensors capable of detecting 8 distinct motion states, including walking, running, and squatting, with high accuracy [105]. The system demonstrated strong linearity and durability across 180,000 compression cycles, making it suitable for continuous gait monitoring and the early detection of conditions such as Parkinson disease, diabetic foot ulcers, and stroke-related asymmetries.

When combined with vibration sensors, the system’s sensitivity to ambient and mechanical cues is significantly enhanced [106]. Vibration sensors can detect oscillatory signals from footsteps, appliance usage, or structural interactions, contributing to a context-aware understanding of the home environment. Vibration sensors are being used in health care for monitoring heartbeats, respiratory movements, and neuromuscular activity, especially when integrated into wearable or ambient systems.

Together, pressure and vibration sensors form a multimodal sensing framework that captures both physical interactions and environmental conditions. These data streams are transmitted via IoT-enabled sensor networks to TEH platforms, where ML algorithms analyze patterns to infer the user’s current health status, detect changes in desired patterns, and support predictive interventions. This integration enables continuous, passive, and personalized health monitoring, particularly for populations at risk of mobility decline or cognitive impairment.

#### Specialized Health Monitoring Tools

Devices such as glucometers, EEG (electroencephalography), and ECG monitors are used for specific health conditions, providing detailed physiological parameters on glucose levels, brain activity, and cardiac function [107,108]. These devices are often part of a broader network of sensors that communicate with a central system to provide a holistic view of the occupant’s health. To ensure efficient analysis, fog computing architectures are often used to deploy ML algorithms close to the data source. This enables low-latency processing for real-time health predictions, reducing reliance on distant cloud servers and supporting timely clinical interventions. Among commercial innovations, AliveCor has developed a Bluetooth-enabled ECG device that allows users to visualize cardiac signals directly on their smartphones. With 6 leads, the device can detect atrial fibrillation within 30 seconds, offering rapid diagnostic support in home settings. EcgMove 4 is another device that can provide real-time ECG data along with other parameters such as acceleration, barometric air pressure, and temperature, which enhances the measurement characteristics to compensate for any disturbances. The data collected through Bluetooth could be easily shared with physicians with the assistance of the TEH network, which assists in predicting a stroke in advance.

### Wireless Connectivity Approaches in TEH Systems

Robust wireless connectivity underpins the operational framework of smart medical devices, allowing for dynamic data exchange, remote health supervision, and integration with broader smart home networks. Short-range communication technologies such as Bluetooth Low Energy (BLE) are instrumental in enabling wearable biosensors and fitness trackers to transmit data efficiently while conserving power, making them ideal for continuous use [109]. For more expansive connectivity, devices are increasingly using Wi-Fi and specialized IoT protocols to synchronize with cloud platforms, enabling advanced diagnostics and data analytics [110]. The introduction of 5G networks further revolutionizes this space by enhancing bandwidth and reducing latency, thereby supporting simultaneous, high-volume data transmission across multiple devices in real time.

Beyond technical capabilities, connectivity must also address data privacy and regulatory compliance, requiring devices to adhere to standards such as HIPAA (Health Insurance Portability and Accountability Act) and the General Data Protection Regulation. This is typically achieved through encrypted communication channels, secure firmware updates, and rigorous authentication protocols. In sum, wireless communication is not merely an enabler of convenience, but it is fundamental to delivering responsive, predictive, and personalized health care within smart living environments.

Wireless communication in wearable TEH devices presents significant power and efficiency challenges due to the complex electromagnetic interactions between the device and the human body. As RF signals propagate through biological layers such as skin, fat, and muscle, they encounter media with distinct dielectric properties, resulting in scattering, absorption, and reflection that degrade transmission quality [111]. To compensate for these losses, devices often require elevated transmission power, which strains limited energy sources such as miniature batteries or energy harvesting modules.

Moreover, dielectric mismatches between wearable materials and biological tissues can disrupt antenna impedance matching, leading to frequency shifts and reduced RF efficiency. These effects are exacerbated by dynamic factors such as movement, perspiration, and posture changes, which introduce temporal variability in signal integrity. Maintaining reliable wireless connectivity under such conditions demands advanced design strategies and material innovations.

### Internet of Medical Things

The proposed TEH network structure is shown in Figure 2. The diagram highlights the mutual benefits distributed across patients, caregivers, and health care providers using the TEH hub as a control unit based on input from biosensors and adaptive ML tools. The Internet of Medical Things (IoMT) represents a transformative paradigm in health care delivery, characterized by the digital interconnection of medical devices, smart home systems, and clinical systems to enhance patient care outcomes [112]. By facilitating the continuous collection and exchange of physiological and behavioral data, IoMT enables early detection of health risks, timely diagnosis, and proactive intervention while simultaneously reducing the burden on health care providers through automated decision support and remote monitoring capabilities.

At its core, IoMT involves transitioning conventional health care models into a digital landscape by enabling low-cost, low-power, low-energy, and low-data transfer with high efficiency and security [112,113]. This evolution, known as interoperability, relies heavily on real-time monitoring technologies and data integration across interconnected devices, including biosensors, wearable platforms, and smart home infrastructure. These systems perform critical functions such as disease surveillance, therapeutic guidance, and personalized treatment modulation. Central to the IoMT framework is the application of ML algorithms for data interpretation. As datasets become increasingly voluminous and heterogeneous, AI analytics enhance diagnostic precision, risk stratification, and outcome prediction. Advanced models not only facilitate accurate classification of patient conditions but also enable adaptive monitoring and predictive modeling for disease progression.

### Detection and Prediction of Disease Within Smart House Through TEH Integration

This section addresses the research objectives 1, 2, and 5 identified in the methodology, focusing on how smart wearable and ambient sensors, combined with ML algorithms, enable early detection and prediction of health conditions. It evaluates the technical performance of predictive models, the quality of input data required, and the potential for personalized monitoring of vulnerable populations within residential environments.

Smart homes enable continuous health monitoring and prediction of potential health problems through the integration of biomedical sensors with computing networks capable of identifying disease progression patterns. It has been shown that using smartphone apps combined with wearables can lead to a positive behavioral outcome in mental illnesses such as bipolar and depressive disorder [114]. Data fusion techniques further enhance the confidence of health trend predictions by integrating multimodal sensor inputs. Disease prediction is achieved through ML algorithms trained on curated datasets, with outcomes heavily dependent on both the type of model and quality of training data. Applications of ML within smart home health care are diverse. For example, dementia can be identified early with the aid of ML models running on remote computing servers [85,115]. The prediction allows early treatment and enhances cognitive well-being for individuals. Support vector machine (SVM) has been used to predict cardiovascular disease using heart sound signals, where estimated heart rate is processed to predict blood pressure and detect disease progression [116,117]. Similarly, SVM-based classifiers support activity recognition for forecasting and preventing chronic diseases [37]. ML models assist in disease classification and prediction from medical images extracted with the assistance of smart closed-circuit television cameras. Monitoring gait is another area where ML models could be deployed for early detection of gait disorders [112]. ML models are capable of estimating ground reaction forces with low error and sensing gait abnormalities that may signal potential neurocognitive disorders. The ML system enables early intervention opportunities for disease progression [118]. Images and facial expressions have been used in the past for patient monitoring for emotion detection, which can indicate diseases such as schizophrenia and depression [119]. Facial expressions are crucial for diagnosing various emotional disorders [120]. TEH systems coupled with ML algorithms can facilitate early intervention and enhance cognitive and physical well-being.

While ML algorithms have shown considerable promise in the early detection and diagnosis of disease within smart home health care systems, several critical limitations persist that affect their reliability and implementation. Foremost among these is the heavy dependence on both the quality and quantity of input data. The effectiveness of ML models hinges on well-curated training and testing datasets; inaccuracies or insufficiencies in these datasets can significantly undermine prediction outcomes. One method is using deep learning (DL) models, which can be powerful in capturing complex patterns but often present interpretability challenges for health care professionals due to their opaque architectures and computational intricacies. Moreover, the deployment of such models is frequently constrained by insufficient data volume, especially in personalized or longitudinal smart home settings. Similarly, despite the applicability of SVMs in high-dimensional classification tasks, they require extensive and clean datasets to achieve high diagnostic accuracy. Feature extraction for SVM-based models is particularly time-intensive, and performance degradation is common when input data is noisy or artifact-laden.

These limitations underscore the need for robust data preprocessing pipelines, the development of explainable AI frameworks, and integration of clinician-oriented decision support tools to ensure safe, reliable, and trustworthy deployment of ML algorithms within TEH systems.

### The Use of Big Data Methods in Smart Home Environments

ML algorithms constitute the computational foundation of any smart home environment, enabling continuous health monitoring, activity recognition, autonomous decision support, and early disease prediction. Supervised learning approaches remain central to TEH analytics, where algorithms such as decision trees, logistic regression models, and k-nearest neighbor classifiers are commonly trained on structured physiological and behavioral datasets to perform diagnostic categorization and generate timely alerting mechanisms for potential health risks [121,122]. To recognize and capture the correct features of locomotion, gait dynamics, and mobility patterns are analyzed using specialized ML models such as SVMs are analyzed to develop fall detection, gait deviation identification, and rehabilitation monitoring systems [123]. These models typically rely on labeled datasets derived from wearable sensors or environmental monitoring devices and are often evaluated using validation strategies such as cross-validation, hold-out testing, or benchmark dataset comparisons to assess classification accuracy and model stability. These models are trained using motion data collected from accelerometers, gyroscopes, or pressure sensors embedded in wearable devices [124-127]. Several studies report promising performance metrics in controlled experimental environments; however, many datasets used for model development remain relatively limited in scale, often consisting of tens to hundreds of participants. Consequently, although these systems demonstrate high classification accuracy under laboratory conditions, their performance in long-term real-world home environments remains an active area of investigation [128,129]. Another developed model known as context-aware methods (CAMs) extends these capabilities by developing a multiplexed sensor system that can consider inputs from physiological, environmental, and behavioral data to improve contextual accuracy. Fog-assisted IoT architectures represent a prominent implementation of CAMs, as they enable distributed analysis of high-priority health signals such as EEG, ECG, and EMG (electromyography) data directly at the network edge. By performing data processing at intermediate network nodes closer to the sensing devices, fog computing enables distributed analysis of high-priority health signals at the network edge. This reduces latency while supporting dynamic, real-time clinical decision-making processes [130]. CAM models can also incorporate environmental and patient-specific historical data to compute personalized health indices, thereby offering a more robust representation of an individual’s physiological status. Furthermore, the layered topology further facilitates simultaneous acquisition and analysis of multiple monitoring elements such as EEG, ECG, and EMG, enhancing the sensitivity and responsiveness of TEH monitoring systems [68].

Another relevant approach used for long-term sleep analysis and anomaly detection using continuous monitoring datasets is known as the multiarmed bandit model [131]. Similarly, patient-state determination algorithms support predictive alert generation by analyzing multiplexed biosensor data to infer deviations from normal physiological states and send indicators with risk levels for further interventions or early-stage disease progression [42]. Big data infrastructures contribute to scalable and efficient processing of the heterogeneous and large volume of health data captured within TEH ecosystems. Distributed computing platforms enable real-time analytics for clinical decision support and behavioral modeling while enabling responsive care solutions [132]. As highlighted in recent public health applications, big data frameworks have been instrumental in infection surveillance, pandemic transmission modeling, and patient triage optimization [133]. They also play a growing role in logistics, such as predictive modeling for vaccine distribution and deployment planning, where analytics can assist in equitable allocation strategies. Within TEH-specific implementations, big data platforms support the integration of ML algorithms capable of detecting disease signatures and performing physiological assessments using distributed computational architectures [134-138]. These architectures enable simultaneous usage of physiological and environmental variables to generate context-aware predictions and early detection outputs.

However, it is important to recognize the challenges faced with the current big data tools and how they are being compensated for. Certain ML techniques such as k-nearest neighbor–based classifiers often present computational burdens that exceed on-device processing capacities within residential IoT ecosystems, limiting their applicability for real-time TEH inference tasks [66]. Furthermore, model robustness is heavily dependent on the fidelity of input data. Sensor noise, missing data, and measurement artifacts can significantly degrade model performance and compromise diagnostic reliability. In response, comparative studies of classification algorithms such as random forestsradio frequency, multilayer perceptrons, SVMs, and Naive Bayes classifiers have demonstrated the potential for high accuracy, including reports of neural network models achieving up to 99% accuracy when classifying key bio signals [139]. Nonetheless, suboptimal feature extraction processes remain a limiting factor for activity recognition, often reducing classification accuracy in less controlled ambient settings. Alternative approaches such as clustering models and probabilistic frameworks, including hidden Markov models, have shown strong performance in modeling heterogeneous health datasets for disease prediction. DL architectures further expand the analytic capabilities of TEH environments. Convolutional neural networks are particularly effective for identity recognition and activity modeling when trained on gait signals from wearable sensors [140,141]. In parallel, recurrent neural networks, including long short-term memory–based variants, have been used for temporal modeling of movement patterns and for detecting sleep changes using audio signals [142]. Recent enhancements in recurrent neural network frameworks have demonstrated improved energy efficiency at the IoT edge, reducing power consumption through optimized data transmission and localized inference pipelines.

The translation of many ML-based monitoring systems from experimental prototypes to clinically validated health care solutions remains limited [143-145]. Many published models rely on datasets that lack demographic diversity, particularly underrepresenting older adults, individuals with chronic health conditions, and populations from low-resource environments [146-148]. This imbalance may introduce bias in model predictions and reduce generalizability across diverse user groups. Furthermore, only a small subset of proposed algorithms has undergone longitudinal validation in real-world smart home deployments or clinical evaluation studies. Consequently, while ML techniques demonstrate substantial potential for enhancing predictive health care capabilities in TEH ecosystems, further large-scale validation, diverse dataset development, and clinical benchmarking are required to ensure reliability, fairness, and real-world applicability.

### Disparities Related to Smart Home TEH Integration

#### Sociodemographic Disparities

A significant concern in the current smart environment landscape is the sociodemographic bias present in existing research. Most studies and technological deployments have been conducted in high-income, predominantly Western nations, resulting in limited representation of low-income countries. This disparity not only marginalizes diverse cultural, economic, and infrastructural contexts but also contributes to a critical knowledge gap regarding the applicability, usability, and scalability of TEH systems across varied global settings [30]. Without inclusive research and development frameworks, the transformative potential of smart home health care technologies risks being inaccessible or ineffective for underserved populations. Addressing these disparities requires participatory design, equitable funding strategies, and localized innovation models to ensure global relevance and impact.

#### Access to Technology

Another profound barrier to equitable TEH integration is the disparity in access to enabling technologies, most notably broadband internet connectivity [149,150]. Individuals residing in rural or underserved regions frequently encounter infrastructural limitations that restrict high-speed internet connectivity, a foundational requirement for deploying telehealth platforms and accessing digital health resources. This persistent digital divide deepens preexisting health disparities by excluding populations that may benefit most from remote monitoring and continuous support systems [151]. Bridging this technological gap necessitates coordinated policy interventions, sustained investments in digital infrastructure, and equitable deployment strategies. Furthermore, inclusive design approaches that consider connectivity constraints during development and deployment are essential to ensure that TEH systems remain accessible, resilient, and scalable across diverse socioeconomic contexts.

#### Resource Allocation

Resource allocation plays a decisive role in shaping the feasibility and deployment of smart home environments. In many low- and middle-income countries, health care budgets are predominantly directed toward combating infectious diseases and addressing immediate public health crises [136]. This prioritization, though contextually justified, inadvertently sidelines the evolving needs of aging populations, whose care could be significantly augmented through smart home technologies. The lack of investment in aging-related infrastructure curtails the implementation of TEH solutions that use mobile networks, sensor-based monitoring, and behavioral analytics, which are key components in supporting independent living and chronic disease management among older generations [152]. As demographic transitions accelerate in low- and middle-income countries, the absence of such infrastructure widens disparities between acute care provision and the sustainable management of chronic, age-related health challenges. Closing this gap demands a strategic recalibration of health policies and funding frameworks, promoting balanced resource distribution that considers long-term population health trends alongside acute disease burdens.

#### Digital Literacy

To ensure effective adoption and usage of TEH systems, digital literacy is a prerequisite. Older adults, as well as frontline health care providers, often face challenges in understanding and navigating the digital interfaces, interconnected sensor networks, and data-driven functionalities that form the backbone of smart health care ecosystems [153-155]. Without adequate training and tailored support mechanisms, the implementation of TEH solutions may falter, resulting in underuse, user frustration, and widening disparities in health outcomes. People with low income and vulnerable racial and gender minority groups will be at a disadvantage [156]. Furthermore, smart health care devices frequently raise complex ethical and safety considerations from data privacy to autonomous decision-making, which must be fully comprehended by end users to foster trust and responsible engagement [157]. Addressing digital literacy gaps requires inclusive educational strategies, user-centric design, and ongoing engagement to empower all stakeholders in the smart health ecosystem.

Digital literacy also extends to health care professionals, not just end users, because it requires the interpretation and management of continuously generated health data within clinical workflows [158]. If not used properly, TEH integration might produce frequent, unfavorable false alerts and will produce urgent overloads from excessive or poorly prioritized alerts and will not support the decision-making structures. Improving digital literacy should be accompanied by structured training in signal interpretation and defined triage processes to ensure that the integration process enhances and develops traditional clinical decision-making and health outcomes.

#### Cultural Acceptance

The perception, usage, and integration of health care technologies are deeply influenced by cultural norms, beliefs, and values, which can vary markedly across communities and regions. These disparities affect not only the willingness of individuals to engage with TEH solutions but also how these technologies are interpreted and applied in daily life. In situations where digital health tools conflict with cultural expectations around privacy, caregiving, or autonomy, their implementation may be met with fear or resistance, limiting their potential benefits. Consequently, cultural misalignment can exacerbate inequities in access and health outcomes, reinforcing structural barriers to widespread TEH uptake [159,160]. To address this, the development of culturally responsive frameworks grounded in participatory design, cross-cultural research, and inclusive policy remains essential for ensuring equitable integration of TEH innovations.

### Enhancements in Health Outcomes With TEH Integration

#### Overview

TEH systems support a shift from reactive to preventive health care, empowering individuals to engage proactively with their health while enabling clinicians to deliver quicker, informed, and relevant interventions. This section explores the measurable benefits of TEH integration that span across health trajectories, functional independence, and overall quality of life. The included studies reported health, usage, and economic outcomes using heterogeneous nonrandomized study designs, including observational analyses, matched-control evaluations, and statistical modeling. The approaches presented in this section are not intended to quantify definite effectiveness or establish causal inference but to categorize and characterize the analytical approaches of outcome evidence.

#### Early Detection of Health Issues

The deployment of IoT sensors within smart homes allows for continuous passive monitoring of residents’ physiological and behavioral data. Subtle deviations such as irregular gait patterns, sleep disturbances, or temperature anomalies can be flagged in real time, enabling early identification of emerging health conditions. This predictive insight facilitates prompt medical intervention, potentially averting critical incidents and improving prognosis.

The integration of TEH systems within smart homes has significantly advanced the early detection of health issues through continuous, passive monitoring of subjects’ physiological data [161,162]. The fusion of multiple sensing devices, including simple parameters such as heart rate, blood oxygen levels, and body temperature, which can be easily measured, enables identification of clear deviations such as irregular gait patterns, sleep abnormalities, or body temperature shifts. Recent studies have demonstrated that combining smartphone applications with wearable devices can lead to better outcomes in many mental health conditions such as bipolar and depressive disorders [163]. Additionally, DL IoT systems have been developed for the remote monitoring and early detection of health problems in home clinical settings [164]. These predictive insights facilitate prompt medical intervention, potentially averting critical incidents and improving prognosis. A particularly valuable approach is the continuous monitoring of health parameters, especially among older adults, whose physiological changes may be subtle yet clinically significant [165,166]. Sustained observation not only improves temporal granularity in data collection but also helps detect fluctuations and early markers of decline, supporting timely interventions [167].

#### Improved Monitoring of Health Indicators

TEH care systems enable granular, ongoing observation of vital parameters, including mobility, heart rate, and medication compliance. Integrating these data streams into a responsive graphical user interface supports caregivers in analyzing trends, comparing baselines, and detecting deterioration patterns [168,169]. Such systems enhance chronic disease management by transforming episodic care into continuous oversight.

#### Enhanced Patient Lifestyle

TEH-enabled smart homes foster psychological well-being by enhancing comfort, autonomy, and social connectedness. Ambient intelligence such as adaptive lighting, mood-sensitive music, and virtual companionship can reduce feelings of isolation, particularly among older adults [170]. These emotionally intelligent environments serve as therapeutic ecosystems, bolstering mental health and elevating the lived experience.

#### Personalized Health Management

By harnessing AI algorithms and individualized health data, TEH platforms deliver personalized interventions aligned with each resident’s condition and preferences. Whether it is monitoring and adjusting medication schedules, recommending tailored physical activity, or adjusting environmental controls, a patient-focused approach ensures care is not only clinically relevant but also contextually meaningful [171]. New studies, as presented in the study by Mora et al [172], show how home-based health monitoring platforms can support better clinical assessment and more efficient personalized intervention planning. This study shows how to initially capture behavioral patterns before a clinician identifies deviations from expected routines in daily activities. This adaptability drives more precise and effective health outcomes while allowing more independent living.

#### Enhanced and Secure Caregiver Accessibility

IoT systems enable seamless communication between residents and caregivers, ensuring that any health concerns are promptly addressed. As many caregivers are challenged in balancing the needs of their loved ones and carrying their own jobs and financial responsibilities, TEH integration offers a supportive environment that can lead to better health outcomes and more independent living [96,173,174]. However, privacy and trust concerns persist, especially with the absence of standardized data formats and the vulnerability of personal health data traversing multiple platforms [112,175-179]. One study investigated the best approach to implement a privacy-aware home care assistance system specifically for older adults living alone that used multiple biosensors and ML tools to support informal caregivers [180]. The platform developed continuously captures daily patterns and feeds them to the model developed to make it adaptable and then can automatically issue alerts when extreme changes have been detected. This enhances the process by which the caregivers are being informed and provides a more secure approach while reducing the need for direct observation.

#### Reduction in Hospital Readmissions

Through anticipatory monitoring and automated escalation protocols, TEH systems help manage acute episodes before they necessitate hospitalization. Data-driven alerts for abnormal activity or vitals support early intervention strategies, reducing unnecessary admissions [86]. This proactive infrastructure contributes to sustained recovery trajectories, easing strain on health care resources while optimizing resident health outcomes. A recent example is a study conducted 2 years ago that illustrates how the use of home digital monitoring devices was able to significantly lower hospitalizations just after 3 months of implementation and emergency department visits at 6 months after implementation [181]. Moreover, another review on telemedicine interventions using telemonitoring instruments has shown a decrease in readmission rates [182].

However, other issues have recently started to arise, such as user acceptance and economic sustainability in the long term, which reduces the effectiveness of TEH systems [183]. That is because patient compliance is by no doubt a critical factor in the success of IoT-based health care technologies, and high costs associated with technology deployment and maintenance also pose barriers to the widespread adoption of TEH systems. Therefore, an evaluation framework should be developed to investigate the risks associated with these issues.

#### Case Study Example

A recent Australian study conducted a home environment that implemented a before-and-after control intervention experiment design to examine health care usage and evaluate outcomes [184]. The observational study included 100 test patients receiving telemonitoring intervention and 137 matched control patients receiving traditional usual care and were followed for 276 days. Outcomes were assessed using observational comparisons and regression-based modeling rather than randomized assignment. The analysis relied on statistical modeling to estimate changes in medical and pharmaceutical expenditure, hospital admissions, and hospital length of stay before and after the start of telemonitoring. Based on projected expenditure trajectories, the study reported modeled reductions in predicted medical expenditure (46.3%), predicted hospital admissions (53.2%), predicted length of stay (67.9%), and reductions in mortality (between approximately 41% and 48%) relative to controls [184]. These estimates are derived from a combination of observed usage data and statistical prediction models rather than from a randomized controlled design. This example demonstrates the analytical approaches being implemented and how matched control groups are considered to objectively obtain realistic estimates of the impact of TEH interventions on potential health and economic outcomes rather than relying on generalization assessments.

## Discussion

### Summary

This review investigates methods of integrating TEH systems in smart homes by looking at the patterns in technological maturity deployed worldwide and how it is reflected in the performance of both ambient and wearable biosensors that rely on AI-driven health analytics. Our findings show that TEH adoption is concentrated in higher-income regions such as Europe and East and Southeast Asia, with emerging initiatives in lower-resource settings. Smart home systems range from foundational automation to fully interconnected intelligent ecosystems, demonstrating varying levels of clinical functionality and predictive capability. Adoption is limited because of interoperability challenges, usability limitations, and gaps in regulatory frameworks, as these key factors influence user acceptance and the long-term sustainability of TEH systems. Additionally, inconsistent reporting on ethical, usability, and regulatory considerations, coupled with limited long-term clinical validation, is restricting future improvements that could enhance effectiveness and wider deployment. This review highlights technological trends, practical implementation scenarios, and research implications for the development of more clinically effective, socially inclusive, and sustainable smart home health care solutions.

### Infrastructure Implementation

#### Overview

Building upon the disparities described previously, this section presents analyzed perspectives on the conceptual, technological, and infrastructure factors that influence the integration of TEH into residential environments. In this section, a wide range of challenges will be systematically addressed to ensure successful implementation, allow scalability, and consider long-term viability.

#### Conceptual Implementation

Despite the promising benefits of TEH within smart environments, achieving full integration remains a complex challenge. Limited computing capacity in residential environments will most likely reduce the efficiency of objectively recognizing and classifying human activities, especially with isolated single sensors that cannot capture complex patterns; yet, evidence suggested that deploying multiple sensors may increase system complexity and intrusiveness [32]. Environmental uniformity in data collection restricts model generalization, making predictive algorithms overly dependent on localized feature sets [33].

System reliability is heavily influenced by infrastructure variables such as Wi-Fi coverage, device upkeep, and the quality of internet services, factors that directly impact data transmission fidelity and monitoring effectiveness [42,134,135,172,180]. Privacy and trust concerns persist, especially with the absence of standardized data formats and the vulnerability of personal health data traversing multiple platforms [112,175-179]. Additionally, the large volume of data generated by diverse sensors can overwhelm communication protocols and resource-constrained hardware, while a lack of interoperability across IoT devices poses a significant barrier to streamlined TEH deployment [168].

Although edge computing enhances real-time processing, its limited storage and computational capacity necessitate reliance on cloud infrastructures, introducing latency and security risks [102,169]. Validation remains a bottleneck for noninvasive and semi-invasive sensors, many of which lack clinical approval for broad deployment [173]. Moreover, disparities in digital infrastructure, especially in rural areas, widen gaps in access, underscoring the digital divide’s role in perpetuating health inequities [159].

Smart environments struggle with long-term activity recognition due to the demand for extensive training datasets, frequent recalibration, and platform dependency, which is particularly challenging for older residents [42,47,174]. Sensor design limitations, such as poor skin compatibility and sensitivity to environmental factors, further compromise data quality [36,91,174]. The absence of clinically validated parameters hampers the translation of wearable data into meaningful health outcomes [122], while gaps in eHealth literacy and technology adoption among marginalized populations constrain inclusivity [36].

Security vulnerabilities from smartphone app integration to sensor network breaches call for robust encryption and energy-efficient security protocols [58,151,160]. Addressing these multifaceted challenges requires interdisciplinary collaboration, rigorous validation cycles, and standardized frameworks to ensure the scalable, ethical, and effective implementation of TEH in smart residential spaces. While these conceptual challenges limit the functionality and inclusivity of TEH systems, their practical implementation is further constrained by technological and infrastructural limitations, as discussed next.

#### Technological and Infrastructure Implementation

Foremost among the obstacles is the heterogeneity of devices and platforms. Variations in OSs, sensor types, manufacturers, and communication protocols create severe interoperability bottlenecks. These discrepancies often result in inefficient data exchange, inconsistent performance, and resource allocation conflicts across devices. While frameworks have been proposed to unify operations, the absence of universally standardized communication protocols remains a persistent issue, impeding seamless integration and real-time data synchronization.

Limited battery capacity of wearable and embedded sensing devices poses another challenge, especially due to continuous data acquisition and computation. Sustainable operation necessitates low-power hardware architectures, adaptive duty-cycling strategies, and energy-aware communication protocols. Although protocols such as BLE offer promise for restricted-power wearable devices, ensuring longer runtime while maintaining adequate data transmission, their efficacy is often challenged by variable user activity, multisensor environments, and the need for uninterrupted monitoring.

The disaggregated nature of IoT ecosystems results in fragmentation, reducing compatibility between devices from different manufacturers and complicating data harmonization. This lack of interoperability and standardization hampers holistic system performance, inflates development costs, and undermines user confidence in TEH solutions [113]. Protocols such as Zigbee (ideal for battery-powered sensor networks) and LoRaWAN (for long-range motion monitoring), BLE (for real-time wearable communication), and Matter offer partial solutions, but require coordinated adoption across the ecosystem. A recent standard established, called the Matter standard developed by the Connectivity Standards Alliance as a unified IP-based smart home connectivity protocol, provides a common framework that enables devices from different manufacturers to operate together and reduces the need for multiple gateways in a way to simplify user integration [150]. It improves interoperability and helps overcome many of the connectivity challenges identified in this review.

#### Ethical, Usability, and Evaluation

The successful implementation of TEH systems in smart homes depends not only on technical feasibility but also on adherence to ethical principles, universal design, and rigorous evaluation standards. These 3 dimensions are deeply interconnected and together determine the reliability, acceptance, and sustainability of smart health care technologies. Initially, the implementation of TEH technologies raises important ethical questions, particularly around privacy and autonomy. For individuals with cognitive impairments, excessive monitoring may compromise personal freedom and psychological well-being. Ethical design frameworks and transparent user consent protocols are fundamental to building trust in these systems.

These ethical issues are amplified by the technical challenge of data management in smart home systems, where personal sensor data creates new vulnerabilities and performance constraints. The transmission and storage of sensitive physiological and behavioral data raise privacy and security risks. Robust encryption schemes and secure communication frameworks are imperative to protect personal information and uphold patient confidentiality [168]. Emerging technologies such as blockchain have shown potential for enhancing data integrity and security within smart home architectures [82], although their integration remains limited and largely experimental. The vast volume of measured data generated by multiple sensors and IoT devices threatens to overwhelm existing communication protocols, leading to latency, packet loss, and bandwidth saturation. Optical fiber-based networks may alleviate bandwidth constraints, yet infrastructure limitations persist. Introducing middleware layers can facilitate data preprocessing, adaptive bandwidth allocation, and selective transmission, thereby enhancing data exchange efficiency and reducing system load.

Furthermore, the everyday usability of TEH systems plays an equally important role in determining user trust and sustainable adoption. Usability remains a critical determinant of TEH-smart home adoption, particularly among older adults who may struggle with system complexity. Designing accessible, intuitive, user-friendly interfaces with inclusive features such as simplified navigation, voice-based controls, and adaptive personalization is essential to foster user acceptance and long-term use. TEH systems must accommodate diverse cognitive and physical capabilities to ensure equitable access and functionality [115]. To do so, technologies with a user-centered design must be supported by robust evaluation frameworks that ensure TEH systems are effective, interoperable, and aligned with international standards.

Evaluating TEH integration within smart homes demands not only clear performance indices but also alignment with internationally recognized standards to ensure consistency, interoperability, and scalability. Key metrics include data exchange efficiency, compatibility rate across sensing devices, response time, error rate, and resource usage. These metrics need to reflect core system capabilities such as responsiveness, reliability, and integration flexibility. However, without a universally agreed framework, comparing performance across heterogeneous TEH architectures remains challenging. There are standards such as IEEE 2951‐2025 that provide structured evaluation methods for smart home devices, assessing intelligence levels across dimensions such as perception, cognition, coordination, and security. Similarly, ISO/TS 37151:2015 outlines performance indicators for smart community infrastructures emphasizing sustainability, resilience, and user-centric design.

Incorporating IEEE and ISO standards provides a range of strategic advantages that support system development and deployment. First, benchmarking consistency becomes achievable, allowing for reproducible and comparable assessments across diverse platforms and architectures. This consistency lays the groundwork for meaningful performance analysis and longitudinal validation. Standards also foster interoperability, enabling seamless integration of heterogeneous devices, sensors, and middleware—a crucial factor in multimodal smart home environments. Furthermore, they inform scalability and optimization efforts by guiding system upgrades and middleware configuration to meet evolving health care demands. Adherence to these standards supports regulatory alignment, ensuring systems comply with international norms for health care delivery and smart infrastructure, which is vital for global deployment and clinical adoption. Together, these benefits underscore the necessity of standards and metrics for evaluation in advancing robust, reliable, and future TEH-smart home technologies. Practical implementations may include adopting IEEE 802.15.4-based protocols for low-power, high-reliability communication, or leveraging edge computing to reduce latency and optimize bandwidth allocation. Looking ahead, future research and development should prioritize the adoption of harmonized metric frameworks grounded in IEEE and ISO guidelines, ensuring that TEH smart home systems mature toward clinically robust, scalable, and globally deployable solutions. Despite growing efforts to standardize performance evaluation in a universal manner, a comprehensive operational framework that integrates technical performance, interoperability, and human-machine interaction (HMI) remains weak. Once developed, this framework is needed to align usability, patient engagement, and system reliability to optimize both adoption and patient health outcomes.

In addition to all the technical challenges mentioned above, structural barriers impact long-term usability and global adoption of TEH integration. Variations in digital infrastructure, literacy, and regulatory maturity levels across regions significantly influence the feasibility of deploying these systems at a larger scale [156]. This is because in more advanced, wealthy settings, there are usually more developed broadband networks, digital health reimbursement pathways, and established regulatory frameworks that facilitate the integration of remote monitoring and assisted health care services, while in lower-income regions, people often face infrastructural limitations, fragmented policy environments, and constrained health care budgets that restrict the deployment of advanced TEH systems. If not deployed efficiently, TEH integration would amplify existing health disparities by disproportionately benefiting populations with greater technological access and digital literacy. While investing in infrastructure and smart personalized system design, TEH solutions have the potential to reduce geographic and socioeconomic barriers by enabling remote monitoring, expanding access to specialist care, and supporting preventive health care delivery in underserved communities.

#### Developing Regulatory Integration Maturity Framework

Earlier in the Results section, the 3 levels of TEH integration maturity were classified and identified as foundational, connected, and intelligent systems. This classification is useful in demonstrating how the integration process progresses simultaneously with the available architectural complexity. However, currently, in most cases, inconsistent assessment and heterogenous criteria are applied when evaluating or categorizing any form of TEH integration without a standardized form of assessment that examines the sophistication and efficiency of a smart system. Current evaluation approaches primarily assess discrete performance metrics such as response time, compatibility rates, accuracy, and energy efficiency, which quantify a form of effectiveness but not in a comparable fashion as part of a unified model that can be used in system development. This was while 1 recent published framework emphasized the need for a responsible deployment of digital health technologies to ensure that system development aligns with clinical effectiveness, accessibility, and sustainability [156]. However, the study lacks quantitative mechanisms for assessing the maturity and integration readiness of smart home health care systems. This variation in assessment tools used to evaluate TEH integration highlights the need for a maturity framework standard with elements capable of assessing readiness across technical performance, interoperability, clinical validation, usability, and scalability. This framework will allow the translation of potential pilot implementations into sustainable, scalable, and efficient TEH integrations. Most importantly, a structured maturity framework can enable the evolution of TEH systems across the different stages from foundational to multimodal clinically intelligent systems. This framework would serve as a regulatory-aligned standard for telemedicine deployment and remote digital therapeutics in smart homes. This is essential when establishing a compliance assessment that considers both system safety and efficiency based on evident improvements in clinical outcomes and quality of life benefits. To visualize the proposed framework, Figure 3 presents a conceptual diagram that illustrates the three layers of TEH integration maturity and how they differ in complexity and functionality. This diagram illustrates guidelines for the system developers or regulatory parties involved to assess and guide implementation strategies.

**Figure 3.**
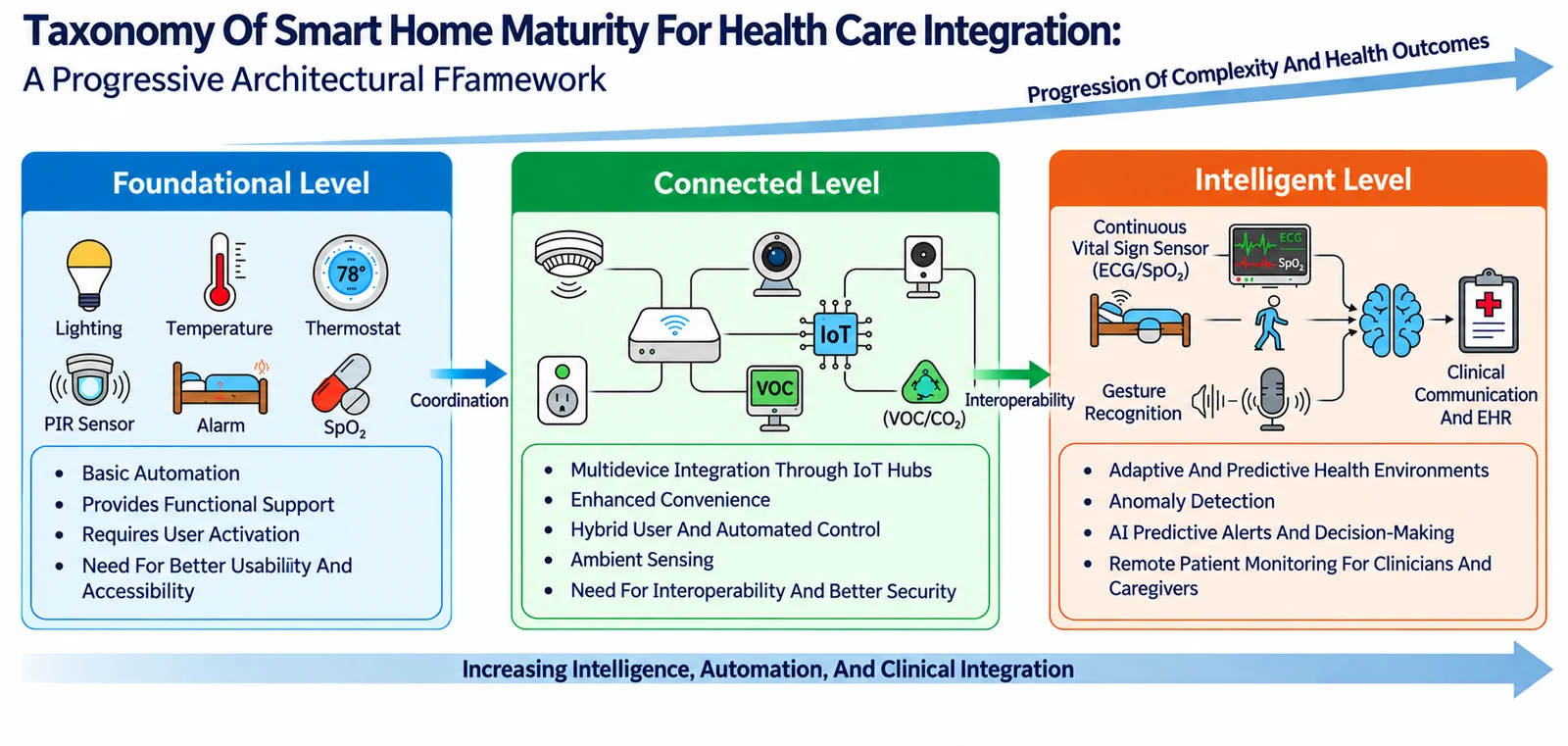
Smart maturity taxonomy of technology-enhanced health care integration: progressive nature of system development, highlighting that as smart home health care technologies evolve, they move from basic operational capabilities into connected systems and then toward more sophisticated, interoperable, and clinically intelligent systems. AI: artificial intelligence; CO₂: carbon dioxide; ECG: electrocardiography; EHR: electronic health record; IoT: Internet of Things; PIR: passive infrared; SpO₂: peripheral capillary oxygen saturation; VOC: volatile organic compounds.

### Future Directions

#### Foundation for TEH Assessments

As ensuring efficient data capture is critical for achieving reliable health insights and predictive outcomes in smart residential environments, it is important to increase participant diversity in empirical studies and address current limitations in sample size and sociodemographic representation. At this stage, it is important to distinguish between near future priorities that will have a direct impact on current TEH systems, including but not limited to improving sensor reliability, data integrity, and its harmony with the different systems involved while there are priorities for the more distant future which will lead the path to scalability and long-term continuous use such as the development of a regulatory-based integration framework, clinical validation, and sustainable widespread expansion. A broader and more representative participant group enhances the generalizability and relevance of findings, ensuring that health monitoring systems remain relevant across diverse populations and cultural contexts. Effective systems must be capable of managing large volumes of heterogeneous data from diverse sensors distributed throughout the smart environment [140]. This includes handling real-time inputs, minimizing latency, and maintaining synchronization across devices.

However, to avoid data redundancy, technical methods are used, such as implementing sliding window-based feature representation, which allows continuous sensor data to be segmented into short intervals, improving temporal precision and reducing redundancy in physiological signal monitoring [185]. Similarly, skeleton extraction techniques translate visual or depth data into simplified human body models, enabling nonintrusive monitoring of mobility, posture, and gait while reducing data volume and privacy risks. These strategies strengthen the accuracy, interpretability, and inclusivity of TEH assessments, forming a critical foundation for future frameworks that integrate sensor reliability, user diversity, and adaptive analytics. Such strategies support the development of comprehensive, resilient, predictive, personalized, and adaptive health care within intelligent living spaces.

#### Potential for TEH Home Integration Frameworks

When approaching long-term needs, more investigative research should be conducted to develop a harmonized regulatory model that factors clinical validation and elements of global-scale integration in health care infrastructures in economically disadvantaged environments. An evaluation metric that looks at and quantifies the level of integration of a TEH device or system is essential to justify its cost of deployment. The next generation of TEH home development should avoid isolated technological innovations and focus on 3 main aspects: sustainable, interoperable, and socially affordable ecosystems. Future research should investigate frameworks that integrate technical scalability, environmental adaptability, policy compliance, and sustainable clinical value.

Additionally, future systems must be evaluated based on their energy consumption to reduce the environmental footprint of continuous sensing. The concept of lifecycle sustainability should extend to software development by adopting modular architectures and open-source frameworks, which can ensure that systems remain adaptable and maintainable across decades of technological evolution. Such an approach supports both environmental sustainability and economic viability, particularly for large-scale deployment in public health infrastructures. Future work should prioritize defining regulatory criteria within integration framework standards that can align and evolve the maturity of TEH systems with tested and validated clinical, social, and economic outcomes.

As TEH systems become increasingly integrated into daily life, HMI must be a central component of future frameworks. Assessing HMI ensures that devices are intuitive, accessible, and adaptable to the diverse needs of users, including older adults and individuals with physical or cognitive impairments. Frameworks should prioritize device flexibility, allowing sensors and wearables to accommodate different body types, living arrangements, and user preferences without compromising accuracy or continuity of monitoring. Equally important is wearability and comfort; continuous monitoring devices, including smart textiles and embedded sensors, must be unobtrusive to encourage long-term adherence. Usability and cognitive load are critical, and interfaces should minimize technical complexity while supporting users with varying levels of digital literacy. Adaptive systems that respond to user routines, updated health parameters, and environmental context can further enhance engagement and reduce burden. For example, a system can look at adjusting sampling frequency in response to detected fatigue or activity levels. Systematic evaluation of HMI within TEH frameworks ensures that devices are not only reliable but also personalized for each case separately. This strategy promotes sustained engagement and effective integration into daily life.

Sustainable TEH frameworks must also integrate a rigorous assessment of ethical, legal, and social implications to ensure alignment with societal norms, patient rights, and regulatory standards. Privacy and data security are foundational, requiring robust encryption, access control, and compliance with internationally recognized regulations and regional standards. Frameworks should also assess autonomy and consent, particularly for vulnerable populations, ensuring that continuous monitoring respects individual choice and maintains user control over data collection and sharing. Equity and accessibility are essential; TEH systems must be evaluated for usability across diverse socioeconomic, cultural, and demographic groups, with attention to adoption patterns and engagement barriers. Transparency and algorithmic accountability are critical for measured biosensor data, as it requires predictive models to be interpretable, auditable, and free from systemic bias. Finally, the social and psychological impact of monitoring technologies, including perceived intrusiveness, trust, and potential effects on mental well-being, should be evaluated. Embedding operational assessment into TEH frameworks will ensure that smart home health care systems are not only technically robust but also ethically sound, socially responsible, and legally compliant, supporting long-term adoption and personalized care. Such frameworks will guide the development of intelligent living spaces capable of delivering predictive, personalized, and equitable health care while remaining scalable, maintainable, and socially acceptable across diverse populations and contexts.

#### Enhancing Adoption in Future TEH Systems

As a near-future target, improving usability, interoperability, and clinical use of TEH systems is essential to improve user engagement and global trust in digital therapeutics and address the challenges faced by clinicians during the integration process. The incorporation of smart sensor data with clinical workflow will require a structured process in prioritizing alerts while assigning clinical responsibility and minimizing excessive or nonactionable notifications. To achieve this, it is critical to evaluate false positive rates, signal specificity, and escalation protocols to prevent unnecessary interventions or patient anxiety. In the long term, this will enable access across diverse deprived populations. Effective integration of TEH in smart homes relies on the seamless and secure transmission of health data from wearable devices and remote monitoring systems into clinical workflows. However, concerns about data privacy and cybersecurity remain significant barriers to adoption. Implementing multilevel encryption strategies alongside robust data governance frameworks is essential to maintaining user trust and ensuring regulatory compliance.

Personalization and usability play a critical role in patient satisfaction. Unfortunately, many wearable devices and smart home sensors lack intuitive interfaces or adaptive features, limiting accessibility for users with diverse health needs, living situations, and levels of digital literacy. Enhancing usability and tailoring system interactions to individual preferences and clinical contexts are therefore crucial for encouraging engagement and sustainable adoption. TEH solutions with intuitive interfaces and personalized features can produce clinical benefits and achieve higher satisfaction ratings. This can be assessed using a multidimensional approach by combining quantitative and qualitative methods. For instance, the general evaluation of medical services questionnaire captures core dimensions including convenience of use, perceived value, efficiency, and overall satisfaction, reflecting both operational improvements and user experience factors. Complementary qualitative approaches, including structured surveys and detailed interviews, provide insights into user perceptions, motivations, and barriers, enabling developers to tailor future iterations of TEH systems. By integrating these quantitative and qualitative evaluations, smart home health care solutions can be refined to maximize usability, trust, and positive outcomes, which lead to more effective and sustained engagement.

### Implications for Research and Practice

Building on the challenges discussed and the perspectives outlined in this section, the work done in this review provides a detailed layout for the process of TEH integration in smart homes, highlighting conceptual, technological, ethical, and infrastructural elements. Unlike previous research reviews published, this work primarily focuses on both technological and clinical outcomes while presenting a holistic picture of how wearable and ambient sensors are linked with health care data analytics, interoperability challenges, and HMI.

Furthermore, another main contribution of this work is that by connecting technical performance with usability, ethical considerations, and scalable deployment strategies, it demonstrates a realistic potential of TEH integrated homes. Its findings guide researchers, developers, and policymakers in designing clinically robust, socially responsible, and accessible smart home health care solutions. Additionally, it supports personalized health management and enhances caregiver accessibility while promoting equitable adoption. This aids in the development of TEH frameworks that are sustainable, inclusive, and capable of improving health outcomes across diverse populations.

### Limitations

Unfortunately, most research on TEH-smart home systems is concentrated in higher-income regions because that is where they can afford to investigate the effectiveness of advanced technologies, which limits generalizability in under-resourced or rural settings where digital infrastructure, regulatory maturity, and health care resources differ significantly and would be more expensive to deploy. The conclusions of this review about deployment feasibility may not extend to low-resource settings.

Another limiting factor for this review is that sample sizes in many studies are small, and again participant sociodemographic diversity is limited, which constrains the applicability of findings across different age groups, cultural contexts, and health conditions. That explains why currently many TEH systems included in the literature lack long-term clinical validation, standardized evaluation metrics, and interoperability across devices, which restricts the robust assessment of effectiveness, scalability, and real-world implementation.

Another limitation faced when presenting this review is how ethical, usability, and regulatory considerations are inconsistently reported across the TEH literature, which significantly limits understanding of how these systems are perceived and adopted by users. Without clear reporting on these issues, it is difficult to assess whether TEH systems respect user rights and maintain trust, which are critical factors for long-term engagement, particularly among vulnerable populations such as older adults or individuals with cognitive impairments. It limits stakeholders’ understanding and confidence in the practical feasibility, scalability, and sustainable adoption of TEH systems in health residential settings (Figure 4).

**Figure 4.**
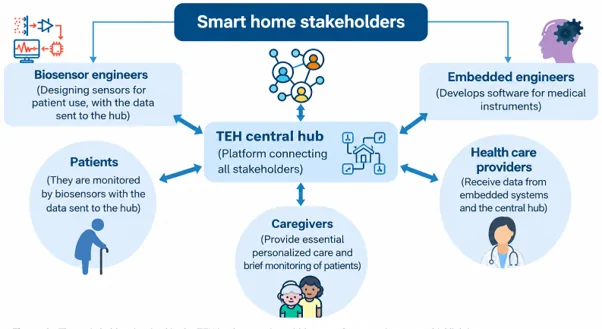
The stakeholders involved in the TEH implementation within smart home environments, highlighting bidirectional communication, interoperability among heterogeneous sensors, and the central role of the TEH hub in coordinating monitoring, decision support, and feedback delivery, as discussed in the literature [25,92]. TEH: technology-enhanced health care.

### Conclusions

Integrating TEH systems into smart home environments is reshaping how health services are delivered, offering continuous monitoring, early detection of risk, and support for independent living. This review demonstrates that multimodal sensor systems that include wearable biosensors, ambient environmental sensors, and specialized medical devices can collectively strengthen chronic disease management and improve clinical outcomes. Studies across Europe, Asia, and the Middle East provide evidence of reduced hospital readmissions, decreased emergency visits, enhanced caregiver accessibility, and improved patient engagement when these systems are properly implemented. The progression from foundational automation to connected and fully intelligent smart home ecosystems reflects increasing maturity in TEH integration and highlights the growing feasibility of personalized, adaptive health care within residential settings.

Despite this progress, this review identifies persistent fragmentation in system design, data governance, interoperability, and clinical validation. Adoption remains uneven across global regions, influenced by variations in digital infrastructure, regulatory readiness, economic capacity, and worldwide cultural acceptance. Although ML applications show great impact in activity recognition, disease prediction, and signal analysis, they will remain limited by data quality, model interpretability, and computational constraints. Wearable devices and ambient sensors also face challenges related to measurement accuracy, sensor placement, packet loss, network reliability, and long-term user adherence. Therefore, technical, infrastructural, and personalization barriers need to be addressed to enable scalability and allow the implementation of TEH smart home systems from pilot studies to sustainable real-world deployments.

Building on the discussion of unified TEH evaluation tools and the interoperability challenges, this review demonstrates the absence of a quantified objective framework model that can assess the TEH integration maturity of any potential system implemented or projected. Current implementations range from simple monitoring elements to highly adaptive environments driven by AI and multimodal sensing platforms, yet there is no consistent method to evaluate readiness, interoperability, clinical reliability, or sustainability. Establishing a structured operational framework that can weigh environmental constraints, technical performance, data quality, privacy safeguards, and long-term scalability is essential for guiding potential implementation. This framework will provide a regulatory standard and a compliance assessment model for telemedicine and digital therapeutics that improves safety standards and health care value. Such a maturity model would enable researchers, policymakers, and health care providers to benchmark and quantify systems, identify gaps, support regulatory approval, and align implementations with measured clinical outcomes and ethical standards.

Addressing broader disparities is equally crucial, as unequal access to broadband connectivity, limited digital literacy, affordability challenges, and sociocultural concerns continue to restrict equitable adoption. Ensuring that TEH home integration reduces rather than deepens existing health inequities requires policies that promote inclusion, accessible design, and personalized training, alongside data governance models that preserve trust and privacy. This review establishes the need for coordinated, quantifiable, and recognized frameworks that can guide the responsible and scalable integration of TEH within smart homes. By addressing existing technical, environmental, and policy challenges and by systematically evaluating implementation outcomes, future TEH ecosystems can support personalized, ethical, and resilient health care delivery.

## Supplementary material

10.2196/89189 Checklist 1 PRISMA ScR Checklist

## Abbreviations

BLE: Bluetooth Low Energy
CAM: context-aware method
DL: deep learning
ECG: electrocardiography
EEG: electroencephalography
EMG: electromyography
HIPAA: Health Insurance Portability and Accountability Act
HMI: human-machine interaction
IoMT: Internet of Medical Things
IoT: Internet of Things
mHealth: mobile health
ML: machine learning
PPG: photoplethysmography
PRISMA-ScR: Preferred Reporting Items for Systematic Reviews and Meta-Analyses extension for Scoping Reviews
SpO_₂_: peripheral capillary oxygen saturation
SVM: support vector machine
TEH: technology-enhanced health care
